# Oncomicrobial vaccines mitigate tumor progression via precisely targeting oncomicrobes in mice

**DOI:** 10.1093/procel/pwae067

**Published:** 2025-01-07

**Authors:** Yudan Mao, Yan Li, Xianzun Xiao, Junrui Mai, Gan Lin, Sheng Liu, Jiayuan Huang, Xiangting Zhou, Xiangyu Mou, Wenjing Zhao

**Affiliations:** Shenzhen Key Laboratory of Systems Medicine for Inflammatory Diseases, School of Medicine, Shenzhen Campus of sun Yat-Sen University, Shenzhen 518107, China; Shenzhen Key Laboratory of Systems Medicine for Inflammatory Diseases, School of Medicine, Shenzhen Campus of sun Yat-Sen University, Shenzhen 518107, China; Shenzhen Key Laboratory of Systems Medicine for Inflammatory Diseases, School of Medicine, Shenzhen Campus of sun Yat-Sen University, Shenzhen 518107, China; Shenzhen Key Laboratory of Systems Medicine for Inflammatory Diseases, School of Medicine, Shenzhen Campus of sun Yat-Sen University, Shenzhen 518107, China; Shenzhen Key Laboratory of Systems Medicine for Inflammatory Diseases, School of Medicine, Shenzhen Campus of sun Yat-Sen University, Shenzhen 518107, China; Shenzhen Key Laboratory of Systems Medicine for Inflammatory Diseases, School of Medicine, Shenzhen Campus of sun Yat-Sen University, Shenzhen 518107, China; Shenzhen Key Laboratory of Systems Medicine for Inflammatory Diseases, School of Medicine, Shenzhen Campus of sun Yat-Sen University, Shenzhen 518107, China; Shenzhen Key Laboratory of Systems Medicine for Inflammatory Diseases, School of Medicine, Shenzhen Campus of sun Yat-Sen University, Shenzhen 518107, China; Shenzhen Key Laboratory of Systems Medicine for Inflammatory Diseases, School of Medicine, Shenzhen Campus of sun Yat-Sen University, Shenzhen 518107, China; Shenzhen Key Laboratory of Systems Medicine for Inflammatory Diseases, School of Medicine, Shenzhen Campus of sun Yat-Sen University, Shenzhen 518107, China

**Dear Editor,**

Colorectal cancer (CRC) is a leading global cancer, causing significant mortality and morbidity, particularly among younger individuals (Spaander et al., 2023). The gut microbiota, including certain bacterial species, has been identified as oncomicrobes and linked to CRC development (Holt, 2023). Oncomicrobes are microorganisms capable of causing cancer and can potentially influence the development and progression of tumors. Research demonstrates oncomicrobes, such as *Fusobacterium nucleatum* (Rubinstein et al., 2013), *Campylobacter jejuni* (He et al., 2019), and enterotoxigenic *Bacteroides fragilis* (ETBF) (Wu et al., 2009), contribute to CRC by causing genetic damage and modulating the immune system. Interventions to modify the gut microbiota, such as broad-spectra antibiotics and bacteriophage therapy, have shown potential in reducing oncomicrobes, which consequentially suppress CRC development. However, the emerging threat of antibiotic resistance and the need for targeted therapies highlight the need for novel approaches. For instance, therapeutic strategies could inhibit colibactin production by polyketide synthase (*pks*^+^) *Escherichia coli* using tungstate to reduce genotoxin-producing bacteria (Cougnoux et al., 2016). Fecal microbiota transplantation has shown promise in restoring microbial balance, but safety concerns and the narrow host range of phages limit their clinical use (Cullin et al., 2021). Vaccines targeting cancer-associated microbes hold promise for preventing and treating cancers, such as Gardasil (Merck & Co, USA), which is the first FDA-approved vaccine for primary human papillomavirus (HPV) and has demonstrated nearly 100% effectiveness in preventing cervical precancerous lesions in previously unexposed individuals (Garland et al., 2016). However, there is a lack of comprehensive investigation into the efficacy of oncomicrobial vaccines in oncomicrobes-associated CRC.

In this study, we used inactivated whole-cell vaccines targeting oncomicrobes as proof-of-concept and demonstrated that oncomicrobial vaccines could mitigate tumor progression. The vaccines specifically reduced the colonization of these oncomicrobes and suppressed tumor development, suggesting a potential for clinical application without disrupting the gut microbiota. Further research is necessary to translate these findings into effective CRC prevention and treatment strategies.

To validate the concept of using vaccines to prevent or treat oncomicrobes associated with CRC, we set up two animal models: preventive and therapeutic. In the preventive model, we used the oncomicrobe *C*. *jejuni,* which is a common exogenous gastrointestinal pathogen that causes bacterial gastroenteritis and various other gastrointestinal diseases and is associated with CRC and promotes tumorigenesis (He et al., 2019), and it is feasible to immunize host before *C*. *jejuni* infection. We prepared a formalin-fixed whole-cell *C*. *jejuni* vaccine. We tested this vaccine in the *Apc*^min/+^ mice model, which carries an adenomatosis polyposis coli (*Apc*) mutation frequently mutated in human colon cancer and increased tumor formation and inflammation in the gut (Ren et al., 2019). Mice received two vaccinations with 10^8^ CFU of formalin-fixed *C*. *jejuni* and challenged with 10^8^ CFU of *C*. *jejuni,* alongside 2.5% DSS to accelerate colonic tumorigenesis (Fig. 1A). We assessed antibody levels against *C*. *jejuni* in serum and feces by Enzyme-Linked Immunosorbent Assay (ELISA). The vaccinated mice exhibited high levels of anti-*C*. *jejuni* antibodies in serum 2 weeks post-booster vaccination (Fig. 1B and 1C), with anti-*C*. *jejuni* total immunoglobulin (Ig) and IgG reached the optimal level a week after the booster vaccination (Fig. S1A and S1B). To investigate how much anti-*C*. *jejuni* IgG was secreted into the intestinal lumen, we measured anti-*C*. *jejuni* IgG levels in mouse feces. As shown in Fig. 1D, the vaccinated mice exhibited significantly higher levels of secreted IgG antibodies in feces than the mock group. Notably, anti-*C*. *jejuni* IgG was not detected in feces until 2 weeks after the booster vaccination (Fig. S1C). To assess the vaccine’s impact on *C*. *jejuni* colonization in the gut, we quantified *C. jejuni* in fecal samples by qPCR using specific primers that amplify the *hip*O gene. *Campylobacter jejuni* was detected in fecal samples of both *C*. *jejuni* and vaccine-*C*. *jejuni* mice at similar levels during the first week after the challenge but decreased in the vaccinated group beyond the first week, with significant differences observed at later time points (Fig. 1E). FISH analysis confirmed these findings on colon samples when mice were sacrificed (Fig. 1F). Thus, the vaccine elicited systemic humoral immunity responses and, although it did not prevent the initial colonization, it did accelerate the clearance of *C*. *jejuni* starting from 1 week after the challenge.

**Figure 1.**
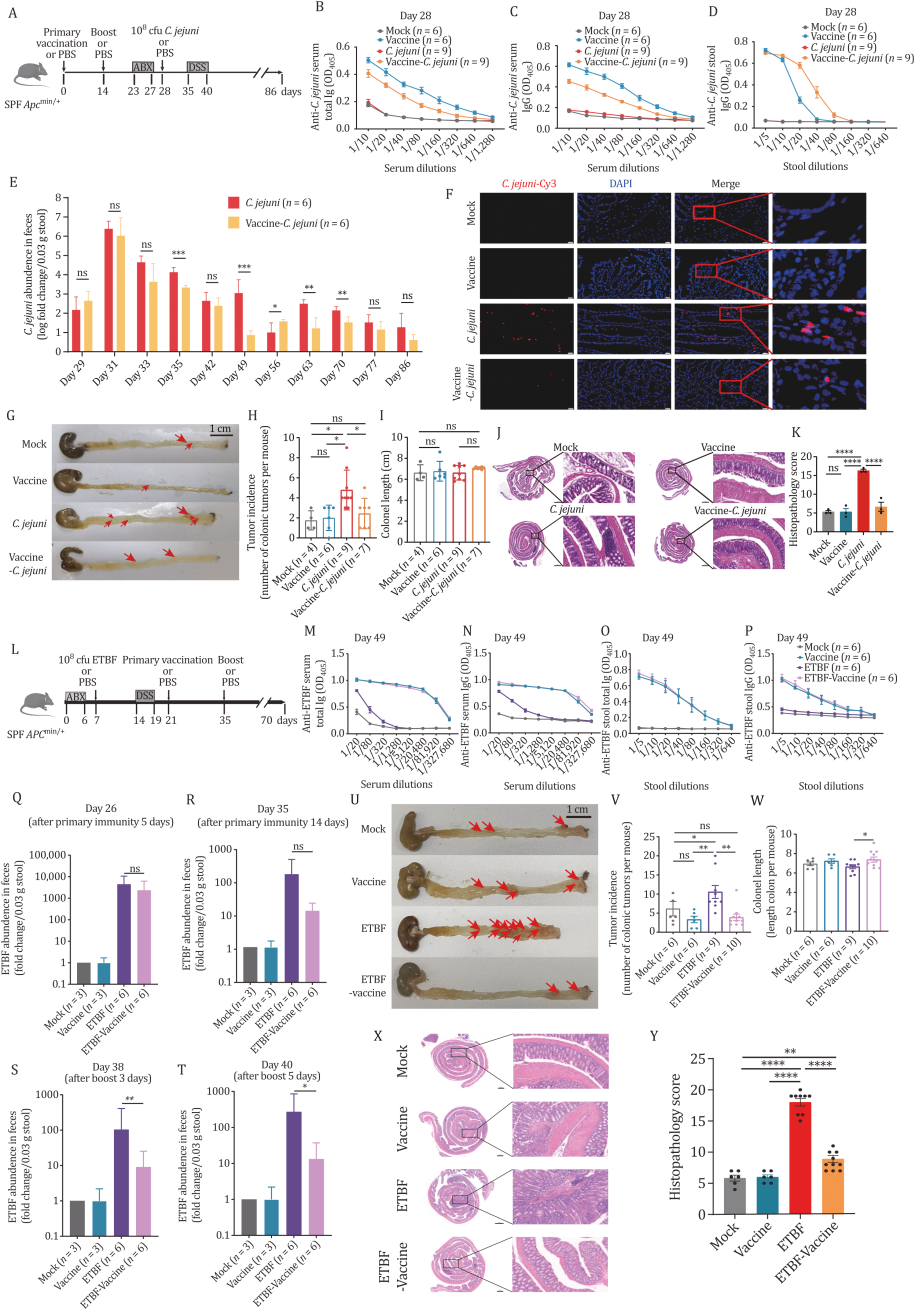
**The vaccines elicit high levels of specific antibodies and effectively reduce oncomicrobes burden and tumorigenesis in preventive and therapeutic models.** (A) Schematic diagram of animal experiments in the preventive model. The schematic diagram shows the experimental design and timeline of the *Apc*^min/+^ mouse model (Mock: *n* = 6, Vaccine: *n* = 6, *C*. *jejuni*: *n* = 9, Vaccine-*C*. *jejuni*: *n* = 9). (B and C) Total immunoglobulin and IgG titers against *C. jejuni* in serum after boosting 2 weeks (day 28) from mock, vaccine, *C*. *jejuni*, and Vaccine-*C*. *jejuni* mice. (D) IgG titers against *C*. *jejuni* in stool after boosting 2 weeks (day 28). Data are representative of two independent experiments. (B–D) Error bars represent mean ± SEM. (E) Comparison of *C*. *jejuni* bacterial burden in the stool from *C*. *jejuni* and vaccine-*C*. *jejuni* in mice. qPCR of *C*. *jejuni* abundance normalized to panbacterial primers targeting the 16S rRNA gene (UNI 16S) in bacterial DNA extracted from feces (*C*. *jejuni*: *n* = 6 mice/group; Vaccine-*C*. *jejuni*: *n* = 6 mice/group). *P-*values by two-tailed unpaired *t-*test, *P < 0.05, **P < 0.01, ***P < 0.001; ns, not statistically significant. Error bars represent mean ± SEM. (F) Representative FISH images of *C*. *jejuni* in colonic sections from Swiss-rolled enteroids. Nuclei are stained with DAPI. Images obtained at 400× magnification, scale bars, 20 μm. (G) Representative images of gross pathology of the distal large intestine. (H) Tumor incidence in the large intestine. Each dot represents one individual animal. Bars represent the geometric mean ± 95% confidence interval. (I) Colon length per mouse. Data from one representative experiment. (J) Representative images of H&E-stained colonic sections of mice. CaseViewer scanned slides and digital images were obtained. Dysplastic lesions were selected and measured. Scale bars, 1000 μm. (K) Combined histopathology score. Bars indicate the median and the interquartile range. (L) Schematic diagram of animal experiments in the therapeutic model. The schematic diagram shows the experimental design and timeline of the *Apc*^min/+^ mouse model (Mock: *n* = 6, Vaccine: *n* = 6, ETBF: *n* = 9, ETBF-Vaccine: *n* = 10). (M and N) Total Ig and IgG titers against ETBF in serum after boosting immunity 2 weeks (day 49) from mock, vaccine, ETBF, and vaccine-ETBF mice. (O and P) Total Ig and IgG immunoglobulin titers against ETBF in stool after boosting immunity 2 weeks (day 49) from mock, vaccine, ETBF, and vaccine-ETBF mice. (Q–T) Comparison of ETBF bacterial burden in the stool from mock, vaccine, ETBF, and ETBF-Vaccine in mice at indicated time points by qPCR. ETBF abundance normalized to panbacterial primers targeting the 16S rRNA gene (UNI 16S) in bacterial DNA extracted from feces. *P-*values by two-tailed unpaired *t*-test, *P < 0.05, **P < 0.01, ***P < 0.001; ns, not statistically significant. Error Bars represent mean ± SEM. (U) Comparison of mock, vaccine, ETBF, and vaccine-ETBF mice tumorigenesis. Representative images of gross pathology of the distal large intestine. (V) Tumor incidence in the large intestine. Each dot represents one individual animal. (W) Colon length per mouse. Data from one representative experiment. Each dot represents one individual animal. (X) Representative images of H&E-stained colonic sections of mice. CaseViewer scanned slides and digital images were obtained. Dysplastic lesions were selected and measured. Scale bars, 500 μm. (Y) Combined histopathology score. Bars indicate the median and the interquartile range. Data from two independent experiments are represented as the mean ± SEM. Specific *n* numbers are indicated in the figure. *P-*values were calculated by one-way ANOVA [(H), (I), (K) (V), (W), and (Y)] with Holm–Sidak for multiple comparisons, *P < 0.05, **P < 0.01, ***P < 0.001, ****P < 0.0001, ns, no significance.

The above results indicate that the vaccine strategy effectively stimulates the mice to produce specific antibodies and eliminates cancer-promoting bacteria. Next, we investigated whether a reduction in the abundance of cancer-promoting bacteria would prevent and treat oncomicrobe-promoting tumorigenesis. As shown in Fig. 1G and 1H, *C. jejuni-*gavaged mice had significantly higher tumor incidence compared to the mock group, indicating the successful reproduction of this previously reported model (He et al., 2019). More importantly, the vaccine-*C*. *jejuni* mice carried significantly fewer tumors than the *C*. *jejuni-*gavaged ones and had no difference from the mock group (Fig. 1H). Furthermore, the vaccine-*C*. *jejuni* mice exhibited a lower inflammation level in the gut compared to the *C*. *jejuni*-gavaged group, as evidenced by the histopathology staining (Fig. 1I–K), as well as the levels of T lymphocyte infiltration, apoptosis, and cell proliferating (Fig. S2A and S2B). To monitor the impact of the vaccine on the safety of the mice, we measured the body weight of the mice during the experiment. The results showed that the vaccine had no significant effect on the body weight of the mice (Fig. S3). These results demonstrated that the vaccine could prevent *C*. *jejuni-*enhanced tumorigenesis by accelerating the clearance of *C*. *jejuni* without obvious safety concerns.

*Campylobacter jejuni* infection, associated with gut barrier disruption DNA damage, contributes to CRC development (He et al., 2019). The bacterium’s invasion triggers inflammation and promotes tumor growth by affecting gene expression. RNA-seq analysis showed that genes affected by *C*. *jejuni* infection in colon tumors and para-cancerous tissues of mice were also partially restored after vaccine treatment (Figs. S4 and S5; Tables S2–5).

Encouraged by the efficacy of the oncomicrobial vaccine in the preventive model, we further investigated this concept in a therapeutic model, which utilized ETBF as a testing oncomicrobe, a gut bacterium found in 100% of late-stage CRC patients, which promotes inflammation and tumorigenesis (Boleij et al., 2015). Since ETBF is prevalent in the human gut, especially among CRC patients, it is unlikely to be vaccinated before its primary infection. Therefore, assessment of vaccine effects after ETBF infection via a therapeutic model is more feasible. *Apc*^min/+^ mice were treated with an antibiotic cocktail and gavaged with ETBF (ATCC 43858) followed by 2.5% DSS administration to induce colonic tumorigenesis. Formalin-fixed ETBF vaccination was administered on day 21, with a booster vaccination on day 35, and mice were sacrificed on day 70 (Fig. 1L). Antibody levels in serum were measured using ELISA. ETBF gavage increased anti-ETBF total Ig and IgG compared to the control (Fig. S6A and S6B). Primary vaccination with ETBF led to significantly higher anti-ETBF total Ig, IgG, and IgM levels in the ETBF-vaccine group compared to the ETBF-gavaged group (Fig. S6C–E), and anti-ETBF total Ig and IgG remained at relatively high levels at the end point of the experiment (Fig. S6F–J). Subcutaneous vaccination boosted the immune response, with significantly higher anti-ETBF total Ig and IgG levels after the booster (Fig. 1M and 1N). Anti-ETBF IgA levels were higher 2 weeks after ETBF gavage, and the ETBF-vaccine group showed higher total Ig, IgG, and IgA levels after primary immunization (Fig. S7A–F). Booster vaccination further increased antibody levels in mouse feces levels (Figs. 1O, 1P, S8A, and S8B), with IgA and IgG titers being >2-fold and ~8-fold higher after the booster, respectively (Fig. S8C–E). These results indicate a robust immune response and anti-ETBF antibodies in the serum and gut lumen.

To evaluate the vaccine’s effect on ETBF colonization in mice, we measured ETBF abundance in feces collected at time points after primary vaccination (from day 26 to 40) by qPCR using primers that amplify the toxin gene. We observed no significant difference in ETBF abundance between the ETBF-vaccine group and the ETBF group at 5 and 14 days after the primary vaccination; however, 3 and 5 days after the booster, the ETBF-vaccine group had significantly less ETBF abundance (Fig. 1Q–T). This was further confirmed by FISH analysis at the end of the experiment (Fig. S8F). The vaccine effectively reduced the abundance of ETBF in the DSS model, indicating the therapeutic potential of the vaccine strategy against gut pathobionts that had colonized the host. Bacteroides is a major genus in the gut microbiota of mice, and it is important to assess the impact of the ETBF vaccine on the levels of Bacteroidetes at the phylum level and Bacteroides levels at the genus level. The ETBF vaccine shows minimal impact on Bacteroidetes and Bacteroides abundance in mouse gut microbiota (Fig. S9A and S9B). Nontoxic *B. fragilis* (NTBF), as a commensal bacterium, has the extraordinary ability to regulate steady-state mucosal immunity and promote the development of systemic immunity (Sears et al., 2014). The study has shown that *Apc*^min/+^ mice treated with NTBF, a non-oncogenic bacterium, do not affect tumor formation (Wu et al., 2009). The ETBF vaccine increased the abundance of NTBF in mice that received both ETBF infection and vaccination compared to those infected with ETBF only (Fig. S9C). These findings suggest that restraining ETBF colonization can restore NTBF abundance, aligning with previous observations of ETBF reducing NTBF abundance (Wagner et al., 2016).

Regarding the therapeutic effect of ETBF vaccines in mice, the ETBF-vaccine group exhibited a significant reduction in tumor numbers compared to the ETBF group (Fig. 1U and 1V). Additionally, since intestinal inflammation induced by ETBF often leads to a shortened intestinal lumen, we measured the length of the mouse colon to assess the extent of inflammation caused by ETBF. Mice infected with ETBF and subsequently vaccinated showed significantly greater colon length than unvaccinated infected mice (Fig. 1W), indicating that the ETBF vaccine effectively mitigated intestinal inflammation. Furthermore, the ETBF-vaccine mice had a less inflammatory gut than the ETBF group, as evidenced by the histopathology staining and scores (Fig. 1X and 1Y). In addition, reduced nuclear staining and milder inflammatory cell infiltration in the colon tissue of the ETBF-vaccine mice were observed compared to the ETBF mice (Fig. S10A and S10B). Additionally, throughout the experiment, especially the vaccine treatment showed no significant effect on the body weight of the mice, which preliminarily demonstrates the safety of the vaccine in mice (Fig. S11). Taken together, these results demonstrated that the vaccine effectively reduces ETBF-promoted tumorigenesis and inflammation in mice.

Vaccines trigger the immune system to recognize and respond to antigens, generating antibodies that specifically target pathogens to prevent infection. Antibodies can neutralize bacteria or their toxins and help clear bacterial infections and they also activate T cells to identify and destroy infected cells. Nonetheless, it is a well-established fact that conventional bacterial vaccines generally fail to activate certain immune cells, including T and B cells. However, the pathogenic bacterium ETBF fosters an immunosuppressive milieu through diverse mechanisms. We postulated that this suppression could be overturned by a vaccine designed to clear ETBF. To assess the ETBF vaccine’s effects on lymphocyte subsets in mouse spleen and tumor tissues, we conducted flow cytometry (Fig. S12A–C). The vaccine and ETBF infection increased CD45^+^ and CD8^+^ T cells but decreased CD4^+^ T cells compared to the mock group in the spleen of mice (Fig. S12B). However, administration of the vaccine after ETBF infection did not significantly affect the immune cell population in the spleen (Fig. S12B). In tumor tissues, following ETBF infection, vaccination significantly increased the number of CD8^+^ T cells compared to the vaccine group (Fig. S12C), indicating a potential break from immunosuppression. Regarding *B. fragilis* enterotoxin, it disrupts gut barrier function and increases inflammation by cleaving E-cadherin and promoting pro-inflammatory cytokine IL-7A production and β-catenin signaling (Wu et al., 2009). ELISA tests of the cytokines in the mouse serum showed that ETBF infection did increase levels of IL-17A in the pro-inflammatory cytokine compared with the uninfected group, the vaccine treatment after ETBF infection reduced IL-17A level to some extent (Fig. S12D), indicating the vaccine may mitigate the pro-inflammatory response caused by ETBF.

The vaccine, designed to target a specific microbe, prompted our interest in its effect on gut microbiota composition. In the preventive model, 16S rRNA gene sequencing on fecal samples revealed no significant alterations in the composition of the gut microbiota between the vaccinated group and the mock group throughout the experiments (days 14–83), as indicated by alpha diversity (Fig. 2A) and beta diversity (Figs. 2B, 2C, and S13). We further analyzed the bacterial abundance of Campylobacter in the 16S rRNA data. The results showed that administering the vaccine effectively decreased the colonization of Campylobacter, although there was no statistical significance in the data (Fig. 2D–E). These results demonstrated that the vaccination prevented the disturbance caused by *C*. *jejuni* infection on the diversity of gut commensals.

**Figure 2.**
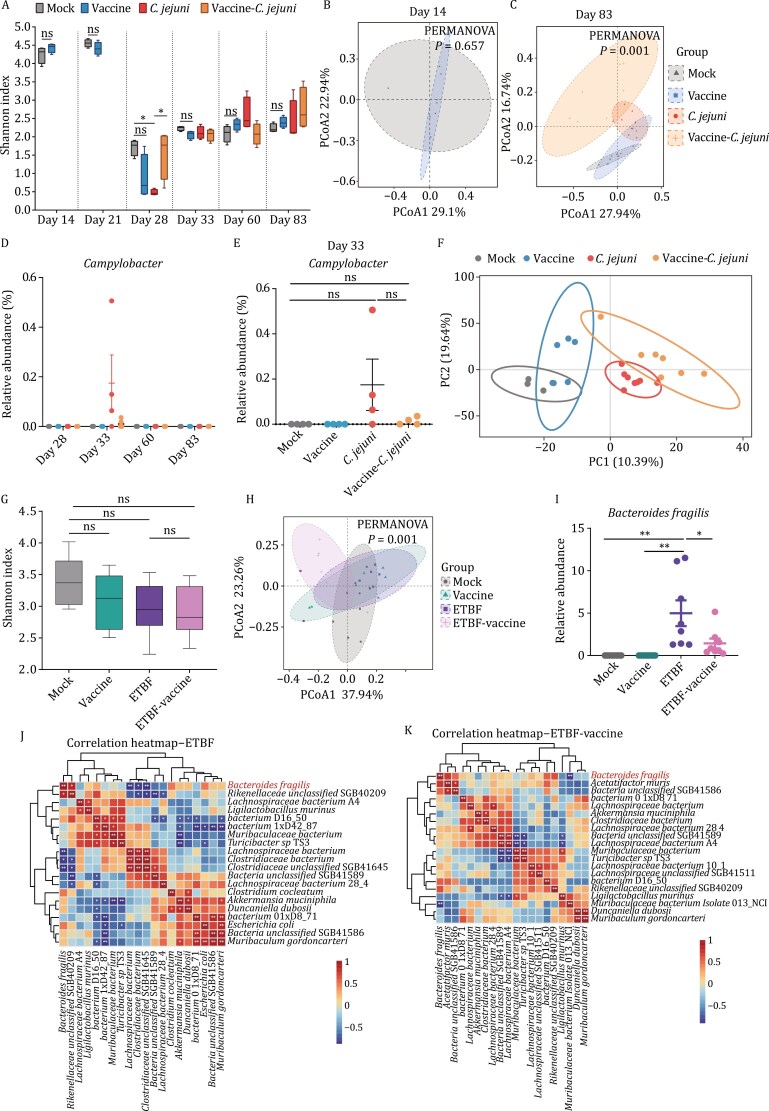
**Vaccine treatment affects oncomicrobe colonization without a major impact on gut bacterial community composition in the preventive and therapeutic model.** (A–E) DNA extracted from the stool of the preventive mouse model receiving indicated treatment was analyzed by 16S sequencing. (A) Alpha diversity. Shannon index at 2 weeks after primary vaccination (day 14), 1 week after boosting (day 21), *C*. *jejuni* challenge before (day 28), after challenge 4 (day 33), 33 (day 60), and 57 (day 83) days. Different colors represent sample groups under different conditions. (B) Beta diversity. PCoA analysis of the microbiota composition (weighted UniFrac distances) at 2 weeks after primary immunization (day 14). (C) PCoA analysis of the microbiota composition after challenge 57 (day 83) days. Different colors represent sample groups under different conditions. (D) Comparison of *Campylobacter* bacterial burden in the 16S sequencing data at *C*. *jejuni* challenge before (day 28), after challenge 4 (day 33), 33 (day 60), and 57 (day 83) days. (E) Comparison of *Campylobacter* bacterial burden in the 16S sequencing data at *C*. *jejuni* challenge after challenge 4 (day 33) days. (F) PLS-DA plot comparison of metabolite profiles from mock, vaccine, *C*. *jejuni*, and Vaccine-*C*. *jejuni* group. *P-*values were calculated using an unpaired two-tailed *t-*test (A) for two-group comparisons and one-way ANOVA [(A), (D) and (E)] with Holm–Sidak for multiple-group comparisons, *P < 0.05, **P < 0.01, ***P < 0.001, ****P < 0.0001, ns, no significance. Error bars represent mean ± SEM. (G–K) DNA extracted from the stool of the therapeutic mouse model receiving indicated treatment was analyzed by metagenomic sequencing. (G) Alpha diversity Shannon index. Different colors represent sample groups under different environments or conditions. Box plots show the interquartile range, median value, and whiskers from min to max. (H) PCoA analysis of the microbiota composition (weighted UniFrac distances). (I) Comparison of *Bacteroides fragilis* bacterial abundance in the metagenomic sequencing data. (J and K) Spearman correlation heatmap of intestinal microflora in mice. (J) ETBF group. (K) ETBF-vaccine group. *P-*values were calculated by one-way ANOVA [(G) and (I)] with Holm–Sidak for multiple-group comparisons, *P < 0.05, **P < 0.01, ***P < 0.001, ****P < 0.0001, ns, no significance. Error bars represent mean ± SEM.

Metabolites derived from gut microbiota play a pivotal role in connecting the microbiome to cancer progression by altering the tumor microenvironment and regulating key signaling pathways (Yang et al., 2023). To explore the effects of a *C*. *jejuni* vaccination on tumorigenesis, we conducted a metabolomics analysis of fecal samples at the end of the study. The analysis revealed that the vaccine did not separate vaccine group and non-vaccine group samples in the PLS-DA model based on multivariate statistical analysis (Fig. 2F). However, there were differences in specific metabolites between vaccinated and non-vaccinated mice (VIP > 1, *P* < 0.05), with the vaccine-*C*. *jejuni* group showed increased levels of antitumor metabolites and decreased levels of tumor-promoting metabolites (Fig. S14A and S14B). The vaccination effectively mitigates the impact of *C*. *jejuni* on the intestinal metabolome, highlighting its potential as an effective intervention.

To probe the vaccine’s effect on the intestinal microbiota’s composition and structure within the therapeutic model, metagenomic sequencing was conducted on mouse fecal samples. The results revealed no significant difference in alpha diversity (Shannon Index) between the mock and vaccine groups (Fig. 2G), and beta diversity analysis also found no significant difference (Figs. 2H and S15A; *P* = 0.084), indicating that the vaccine did not disturb the normal gut microbiota (Fig. S15). Taxonomy composition comparison revealed that ETBF infection increased the number of *B. fragilis* species compared with the other groups (Fig. S16A), but the vaccine significantly reduced *B. fragilis* abundance in the ETBF-vaccine group compared to the ETBF group (Figs. 2I and S16A), aligning with previous qPCR results. In addition, the highest abundance of other species of Bacteroides, such as *Bacteroides caccae*, *Bacteroides ovatus*, and *Bacteroides salyersiae*, remained unchanged (Fig. S16B–D), suggesting that the ETBF vaccine did not have a significant impact on the normal intestinal flora of *Apc*^min/+^ mice.

*Bacteroidetes* play a crucial role in maintaining intestinal health, including nutrient metabolism, microbial synthesis, and immune regulation (Wexler, 2007). Therefore, we urgently need to know whether the ETBF vaccine affects the interactions *B*. *fragilis* between and different microorganisms. To further investigate the impact of ETBF infection and the vaccine on the intestinal microbiota of *Apc*^min/+^ mice, we conducted a correlation network analysis of the microbial community at the species level. The ETBF vaccine preserved the coexistence dynamics between intestinal bacterial species (Fig. S17A and S17B) and some of the functional pathways associated with *B*. *fragilis* were attenuated in vaccine-treated mice following ETBF infection compared with mice infected with ETBF alone (Fig. S17C and S17D). Interestingly, in mice infected with ETBF, some of the *B*. *fragilis*-associated functional pathways showed a significant negative correlation with species of *Lachnospiraceae*. However, in mice treated with the vaccine after ETBF infection, these functional pathways showed a positive correlation with species of *Lachnospiraceae* (Fig. S17C and S17D). The above results suggest that the ETBF vaccine may influence the *B*. *fragilis*-related functional pathways, inhibiting ETBF growth and suppressing tumor development. In contrast, vaccine treatment mitigates the effects of ETBF infection on the rest of the microbiota.

Correlation analysis of the species-level gut microbiota revealed the complex relationship between ETBF infection, the ETBF vaccine, and the gut microbial species in *Apc*^min/+^ mice (Fig. 2J and 2K). In ETBF mice, *B*. *fragilis* positively correlated with *Rikenellaceae bacterium* and negatively correlated with *Lachnospiraceae bacterium* and *Clostridiaceae bacterium* (Fig. 2J), while in ETBF-vaccinated mice, *B*. *fragilis* only negatively correlated with *Muribaculaceae* and positively correlated with *L*. *bacterium* (Fig. 2K). Lachnospiraceae, typically beneficial in healthy gut microbiota, is involved in carbohydrate metabolism (Takeuchi et al., 2023). These findings indicate that the ETBF vaccine reversed the dysbiosis caused by ETBF infection and increased the abundance of beneficial bacteria, such as *L*. *bacterium*.

In conclusion, we assessed the effectiveness of bacterial vaccines in the prevention and treatment of CRC induced by oncomicrobes, specifically *C*. *jejuni* and ETBF. Our findings demonstrate that the vaccine treatment successfully decreased the abundance of specific oncomicrobes, spares gut commensal, and effectively controlled tumor growth in both mouse models. Furthermore, if a vaccine proves to be safe and efficient in ameliorating the occurrence and progression of CRC, it would be worthwhile to explore additional applications. For instance, individuals who are under surveillance for colorectal cancer due to their family history could potentially benefit from receiving cancer-promoting bacterial vaccination.

## Supplementary Material

pwae067_suppl_Supplementary_Figures_S1-S17_Tables_S1-S5

## Footnotes

We thank Dr. Jiaxin Zou and Dr. Hongda Zhang for assistance in the sample collection, Dr. Changhai Ding and Dr. Xiaoshuai Wang for support in the sample collection, Jing Yu for technical support and assistance with animal experiments, and Dr. Peijie Li for bioinformatics analysis.

This work has been supported by the National Key Research and Development Program of China (Grant No. 2020YFA0907800), the National Natural Science Foundation of China (Grant No. 32000096), the Shenzhen Science and Technology Programs (KQTD20200820145822023, ZDSYS20220606100803007 and RCJC20231211085944057).

The authors declare no conflict of interest.

All animal experimental procedures were performed in accordance with the animal protocol (Approval No. 2022003) approved by the Guangzhou Institute of Biomedicine and Health Research of the Chinese Academy of Sciences. The authors declare their agreement to participate and to publish.

Y. M., X. M., and W. Z. conceived and designed all the experiments. Y. M., Y. L., and X. X. performed and analyzed the results of the experiments. G. L. and J. M., participated in the implementation of animal experiments. S. L., J. H., and X. Z. carried out the analysis on 16S, metabolism, and RNA-Seq. Y. M. drafted the manuscript with the assistance of X. M. and W. Z. X. M. and W. Z. reviewed the results and the manuscript. All authors have read and agreed to the published version of the manuscript.

All data generated or analyzed during this study are included in this article. All the custom codes are available from the corresponding authors upon reasonable request.
