# Supplementary material for: Oncomicrobial vaccines mitigate tumor progression via precisely targeting oncomicrobes in mice
Source: Protein Cell. 2025 Jan 7;16(8):724–31. doi: 10.1093/procel/pwae067 (PMC12342133; doi:10.1093/procel/pwae067)
Supplement: pwae067_suppl_Supplementary_Figures_S1-S17_Tables_S1-S5 [file pwae067_suppl_supplementary_figures_s1-s17_tables_s1-s5.pdf]

Supplementary Information

Oncomicrobial vaccines mitigate tumor progression via precisely targeting oncomicrobes in mice

MATERIALS AND METHODS

Ethics statement.

This study involving animal models was conducted in accordance with ethical guidelines and regulations. All animal experiments were conducted in compliance with the National Institute of Health Guide for the Care and Use of Laboratory Animals. The experimental protocols and animal handling procedures were approved by the Animal Ethics Committee at the Guangzhou Institute of Biomedicine and Health Research of Chinese Academy of Sciences (Approval No. 2022003).

Bacterial strains and vaccine preparation.

The human clinical isolate *C. jejuni* strains 81-176 were previously described [1]. The *C. jejuni* 81-176 wild-type strain was cultivated on tryptone soya agar (TSA) plates with Karmali supplement (Oxoid) and 5% fresh sheep blood in a microaerobic atmosphere (5% O_2, 10% CO_2, 85% N_2) at 37 °C for 48h. Karmali supplement was added according to the instructions of the manufacturer. The bacteria were harvested, suspended in 1% formalin for 1 hour, pelleted at 3000 rpm, and washed twice in sterile PBS at 4°C before use.

The bacterial strain ETBF (ATCC43858) [2], obtained from American Type Culture Collection (ATCC), was cultured at 37°C with a gas mix of 5% H_2, 10% CO_2, and 85% N_2 under anaerobic conditions in brain-heart infusion (BHI) broth supplemented with 5% fresh sheep blood. The bacteria were harvested, suspended in 1% formalin for 1 hour, pelleted at 3000 rpm, and washed twice in sterile phosphate-buffered saline (PBS) at 4°C before use.

Immunization and challenge with *C. jejuni* in the DSS mouse model

5-to 8-week-old female *Apc*$^{min/+}$ mice (C57BL6/J background) were randomized and assigned to 4 groups before immunization: mock (*n* = 6), Vaccine (n=6), *C. jejuni* (n=9) and Vaccine-*C. jejuni* (n=9) groups. All mice were maintained in microisolator cages in a specific pathogen-free (SPF) animal facility. Mice in vaccine and vaccine-*C.*

jejuni groups received intraperitoneal injections of formalin-fixed *C. jejuni* (10^8 colony forming units, CFU, in 100 μl of PBS) as the primary immunization. For the mock and *C. jejuni* groups, mice were injected intraperitoneally with PBS. Two weeks after the primary immunization, mice in the vaccine and vaccine-*C. jejuni* groups received intraperitoneal injections of formalin-fixed *C. jejuni* as a boost (10^8 CFU in 100 μl of PBS). For the mock and *C. jejuni* groups, mice were injected intraperitoneally with PBS. Serum samples were collected via tail bleeding 1 week and 2 weeks after the primary and booster immunization for antibody concentration determination.

To induce *C. jejuni* colonization, all mice were administered an antibiotic cocktail (100 mg/L vancomycin, 200 mg/L metronidazole, ampicillin, and neomycin) in their drinking water for 5 days and then discontinued[3, 4]. Mice in *C. jejuni* and vaccine-*C. jejuni* groups were orally challenged with *C. jejuni* 81–176 via gavage (10^8 CFU/mouse in 200μl PBS). In contrast, mice in mock and vaccine groups were treated with PBS. Stool samples were collected before *C. jejuni* challenge and then at 1, 3, 5, and 7 days afterwards. One week after the challenge, all mice were given 2.5% dextran sulfate sodium (DSS, molecular weight 40 kDa, Alfa Aesar) in their drinking water for 5 days and then discontinued. Fecal samples were collected once a week following the DSS treatment.

Six weeks after the DSS treatment, all mice were humanely euthanized by isoflurane. The colons were carefully opened lengthwise, and the visible tumors were counted. The size of the tumors was measured using an electronic digital caliper (Control company). Snips of approximately 0.5 cm × 0.5 cm were taken from the distal colon, rapidly frozen in liquid nitrogen, and stored at −80°C. The remaining colonic specimens were Swiss-rolled, fixed in formalin, and embedded in paraffin for histopathology evaluation (H&E), immunohistochemistry (IHC), and Fluorescence in situ hybridization (FISH). Sections of 5 μm were stained with H&E.

ETBF infection and immunization in DSS mouse model

Apc$^{min/+}$ mice (5~8 weeks old, female, C57BL6/J background) were randomly

assigned to 4 groups prior to infection: mock (*n* = 6), vaccine (n=6), ETBF (n=9), and ETBF-Vaccine (n=10) groups. All mice were housed in microisolator cages in an SPF animal facility. To establish ETBF colonization, all mice were given an antibiotic cocktail (100 mg/L vancomycin, 200 mg/L metronidazole, ampicillin, and neomycin) in their drinking water for 5 days, and then the treatment was discontinued[5, 6]. Mice in ETBF and ETBF-vaccine groups were orally infected with ETBF via gavage (10^8 CFU/mouse in 200μl PBS). For the mock and vaccine groups, mice were treated with PBS. Stool samples were collected before ETBF infection and at 1, 3, 5, and 7 days post-infection to confirm ETBF colonization. One week after ETBF infection, all mice were given 2.5% DSS in their drinking water for 5 days, and then the treatment was discontinued. Mice in the vaccine and ETBF-vaccine group were injected abdominally formalin-fixed ETBF (10^8 CFU in 100 μl of PBS) as a prime immunization. For the mock and ETBF groups, mice were injected with PBS. Two weeks after the prime vaccination, mice in the vaccine and ETBF-vaccine groups were given a boost of formalin-fixed ETBF. For the mock and *C. jejuni* group, mice were injected with PBS. Serum samples were collected via tail bleeding 1 week and 2 weeks after the prime and boost immunization to determine antibody levels.

Five weeks after the boost treatment, all mice were humanely euthanized by isoflurane. The colons were longitudinally cut open and macroscopic tumors were counted. Tumor size was measured using an electronic digital caliper. Snips of approximately 0.5 cm × 0.5 cm were taken from the distal colon, snap frozen in liquid nitrogen, and stored at −80°C. The remaining colonic tissue was Swiss-rolled, formalin-fixed, and paraffin-embedded for H&E, IHC, and FISH.

Enzyme-linked immunosorbent assays (ELISA)

ELISA was used to measure antibody levels in the sera and fecal samples of the mice. For the Sera ELISA, a mouse total Ig ELISA kit (Abcam, ab102448), a mouse IgG ELISA kit (Abcam, ab6728), and a mouse IgA ELISA kit (Abcam, ab97235) as previously described [7]. To coat the ELISA plate with fixed microbiota (*C. jejuni* or

ETBF), 0.5% formalin was used to fix the microbiota for 2 hours. The fixed microbiota was washed twice with 50 mM NaCarbonate (PH9.6) (3.03g Na2CO3, 6.0g NaHCO3 in 1L water). The fixed microbiota (100 μl) was added to the NUNC Maxisorp ELISA plate (Thermo Fisher, 44–2404-21) and incubated at 4°C overnight. The bacteria-coated plate was washed with PBST (0.05% Tween20) three times. The wells were blocked with 5% nonfat milk in PBST buffer (100 μl) and incubated at room temperature (RT) for 2 hours on a gentle shaker. The plate was washed with PBST four times. For serum dilutions, 2% nonfat milk in PBST (90 μl) was added to each well, followed by adding 10 μl of serum to each well as replicates. A 2-fold serial dilution (1/10, 1/20, 1/40, 1/80, etc.) was performed, and the plate was incubated at room temperature for 2 hours. The plate was then washed with PBST four times. The secondary antibodies used were anti-mouse immunoglobulin–horseradish peroxidase (HRP), IgG-HRP, or IgA-HRP at a dilution of 1:2000 in PBST 1% milk (50 μl). The plate was incubated at RT in the dark for 1 hour. After washing the plate with PBST four times, substrate TMB (50 μl) was added to each well and incubated at room temperature for 15 minutes. The OD_{405} was measured using a BioTek Synergy HT Multi Mode Microplate Reader.

For the measurement of fecal antibodies, fecal pellets were weighed and homogenized in 1 mL of PBS and centrifuged, and only the supernatants were used for ELISA. The subsequent steps were similar to the serum ELISA. 2% nonfat milk in PBST (50 μl) was used for fecal dilutions, and 50 μl of fecal supernatants were added to each well as replicates. A 2-fold serial dilution (1/5, 1/10, 1/20, 1/40, etc.) was performed, and the plate was incubated at room temperature for 2 hours. The results were read using a BioTek Synergy HT Multi Mode Microplate Reader at OD_{405}.

Identification of bacterial colonization in fecal samples

The abundance of bacteria in feces was assessed using a quantitative PCR (qPCR) assay with specific primers. Briefly, fecal samples were collected from *Apc*$^{min/+}$ mice and stored at -80°C before DNA extraction. The stools were homogenized and serially diluted in sterilized PBS. Fecal DNA was extracted using the TIANamp Stool DNA Kit, following the manufacturer's instructions. The qPCR assay was performed using the

TB Green Premix Ex Taq (TAKARA BIO INC.) on an ABI Step One Plus Real-Time PCR System. The abundance was then normalized to universal bacterial primers targeting the 16S rRNA gene (UNI 16S)[8]. The relative abundance was calculated relative to the Mock group and Vaccine group. The primer sequences used for bacterial identification are listed in Table S1.

Histopathology evaluation (HE)

Colons were harvested for histology by bisecting longitudinally and prepared as 'Swiss-rolls'. The Swiss rolls were fixed in 4% paraformaldehyde at room temperature for 48 hours. Sections of the Swiss rolls of the colon were cut at a thickness of 3-4 μm and stained with hematoxylin and eosin. The extent of inflammatory changes was determined using the scoring system proposed by Cooper et al[9]. Histopathological examination and scoring were performed by an expert pathologist. Inflammation was scored on a scale of 0 to 4, with 0 representing normal mucosa and 4 representing severe inflammation [6, 10-12].

Fluorescence In situ Hybridization (FISH)

Bacterial colonization in colonic tissue sections was visualized using the FISH assay, as previously described [13]. Paraffin-embedded tissues were sectioned to a thickness of 5 μm. The deparaffinized tissue sections were then incubated in a lysozyme solution, washed, and hybridized with the FISH probe overnight. The specific probe used for detecting the presence of *C. jejuni* was Cy3-tagged with the sequence 5'AGCTAACCACACCTTATACCG3'. The probes were used at a concentration of 5 ng/μl in a pre-warmed hybridization buffer (0.9 M NaCl, 20 mM Tris pH 7.4, 0.01% SDS). The slides were incubated at 55°C in a humid chamber for 90 minutes, washed two times at 55°C in pre-warmed washing buffer (0.9 M NaCl, 20 mM Tris pH 7.4), and then mounted and counterstained with DAPI (contained in the VECTA SHIELD mounting medium). All the probes were manufactured by Sangon. Confocal images

were acquired using a Leica DMi8 confocal microscope with a HCX PL APO 40X (NA 1.25) oil immersion objective.

Immunohistochemistry（IHC）

IHC analyses were conducted on sections of colonic Swiss-rolls that had been immediately fixed in 5% formalin and embedded in paraffin, following the previously described methods[13, 14]. In brief, the colonic tissue sections were deparaffinized, rehydrated, and subjected to antigen retrieval by boiling in citrate buffer. Subsequently, the sections were stained with primary antibodies against cleaved caspase 3 (Servicebio, GB11532, 1:500) to detect apoptotic epithelial cells, Ki67 (Servicebio, GB111499, 1:500) to detect proliferating epithelial cells, F4/80 (Servicebio, GB11027, 1:500) to detect macrophages/monocytes, CD3 (Servicebio, GB111337, 1:500) to detect T lymphocytes, and CD45 (Servicebio, GB11066, 1:1000) to detect B lymphocytes. Secondary antibodies were used for detection as previously described. Positively stained cells were examined under a light microscopy (magnification 100× and 400×), and the average number of respective positively stained cells was determined within at least six high power fields (HPF, 0.287 mm^2, 400× magnification) for each mouse. This analysis was performed by an independent investigator using blinded samples.

16S rRNA gene sequencing and analysis

Mouse stool samples were collected, and fecal DNA was extracted as previously described [15]. The V3-V4 hypervariable region of the 16S rRNA gene was amplified using primer pair 338F (5′-ACTCCTACGGGAGGCAGCAG-3′) and 806R (5′-GGACTACHVGGGTWTCTAAT-3′). Both the forward and the reverse primers contained universal Illumina paired-end adapter sequences, as well as unique individual 4 to 6 nucleotide barcodes between the PCR primer sequence and the Illumina adapter sequence, allowing for multiplex sequencing. The amplicons were purified and then subjected to multiplex sequencing on an Illumina MiSeq platform. The raw sequencing

data were analyzed using QIIME2 pipelines [16]. The resulting feature table of the gut microbiota was utilized for alpha and beta diversity analysis, as well as taxonomic analysis and differential abundance testing. Principal Coordinate Analysis (PCoA) was conducted to derive principal coordinates and effectively visualize variations among samples in intricate multidimensional data.

Untargeted Metabolomics and analysis

Untargeted metabolomics analysis was conducted following previously reported methods. Briefly, 25 mg stools were weighed and extracted using a precooled extraction reagent (methanol: acetonitrile: water, 2:2:1, v/v/v). Internal standards were added for quality control of sample preparation. The samples were homogenized using a TissueLyser (JXFS TPRP, China) for 5 minutes, followed by sonication for 10 minutes and incubation at -20 °C for 1 hour. After centrifugation at 25000 rpm for 15 minutes at 4 °C, the supernatant was transferred for vacuum freeze drying. The metabolites were resuspended in 10% methanol, sonicated for 10min at 4 °C, and then centrifuged at 25000 rpm for 15 minutes. The supernatants were transferred for LC-MS analysis. A quality control (QC) sample was prepared by pooling the same volume of each sample to assess the reproducibility of the LC-MS analysis. Non-target metabonomics analysis was performed by LC-MS/MS, and high-resolution mass spectrometer (Q Exactive, Thermo Fisher Scientific, USA) was used to collect positive and negative ion data for improving metabolites coverage. Compound Discoverer 3.0 software (Thermo Fisher Scientific, USA) was employed for LC-MS/MS data processing, including peak extraction, peak alignment, and compound identification. The Metax software package [17], along with the metabolome information analysis process, was used for data pre-processing, statistical analysis, metabolite classification, and functional annotation. PCoA to reduce the dimensionality of multivariate raw data, allowing for analysis of grouping, trends, and outliers within and between sample groups. The differential metabolites were screened using the VIP values from the first two principal components of the Partial Least Squares Method-Discriminant Analysis (PLS-DA) model,

combined with the results of fold change and Student's *t*-test obtained from univariate analysis.

Mouse RNA-seq and Analysis

The colon tumor and para-cancerous tissues of mice were collected, and tissue RNA was extracted as previously described. The RNA library was prepared and sequenced by the BGISEQ sequencing platform. The quality filtering of reads was performed at Q20 and the remaining adaptors were trimmed using Trimmomatic version 0.36 [18]. The resulting reads were aligned to Illumina iGenome Mus musculus Ensembl GRCm38 reference genome using Bowtie2 version 2.3.0 [19]. The resulting alignments, averaging 21,051,145 reads per sample aligned uniquely to mouse transcriptome, were processed using Cufflinks version 2.2.1 [20]. The precise counting of reads was performed using the featureCounts software[21], quantifying the expression level of each gene in each sample based on the calculation formulas of Fragments Per Kilobase per Million (FPKM) and Transcripts Per Kilobase per Million mapped reads (TPM). The DESeq2 software [22] was used to calculate differentially expressed genes based on the read counts of the samples, with the condition of Q value ≤ 0.05 or FDR ≤ 0.001. The expression levels of genes in different samples were visualized using the R package (version 4.1.3) to generate a clustering heatmap. Gene Ontology (GO) and Kyoto Encyclopedia of Genes and Genomes (KEGG) [23] enrichment analysis were also performed on the differentially expressed genes.

Metagenome sequencing and analysis

Mouse stool samples were collected, and fecal genomic DNA was extracted with the QIAamp fast DNA stool mini kit (Qiagen), as previously described. Filtering of Illumina sequencing adapters and low-quality reads was performed using fastp (version 0.20.0) with default parameters. The removal of host reads was accomplished by aligning the reads to the C57BL/6 mouse reference genome with Bowtie2 (version 2.4.1)

[19]. The composition of the microbial community was determined using MetaPhlAn (version 4.0.2) with default parameters [24]. Gene families were annotated using UniRef90, and MetaCyc pathways were annotated using HUMAnN (version v3.0.0) [25]. Subsequently, quantile normalization and statistical analysis were conducted. Metabolic enzymes and biological functional classifications for pathways were obtained from the MetaCyc website (https://metacyc.org/). The α-diversity of each sample, based on the Shannon index, was estimated. Principal Coordinate Analysis (PCoA) was used to visually assess the differences and similarities between bacterial communities (β-diversity). To determine the similarities and differences between samples, Permutational multivariate analysis of variance (PERMANOVA) analysis was performed using the “adonis” function in the “vegan” package. Differential abundance of species and functional pathways were tested by two-tailed Wilcoxon rank-sum test. P values were adjusted by the Benjamini-Hochberg correction for multiple tests if necessary. Perform correlation clustering, correlation network analysis, and heatmap analysis using OmicStudio tool (https://www.OmicStudio.cn).

Measurement of serum cytokine levels

The levels of mouse interleukin 17A (IL-17A) and tumor necrosis factor alpha (TNF-α) in mouse serum were measured using the mouse IL-17A ELISA kit (Ruixin biotech, China, RX203066M) and mouse TNF-α ELISA kit (Ruixin biotech, China, RX202412M), respectively. The substrate color reaction was measured at 450nm using a microplate reader and then the results were quantified using standard curves.

Flow cytometric analysis

Mouse spleens and tumor tissue were collected, washed with PBS for flow cytometric analysis. Spleen samples were enzymatically processed using 0.05% Collagenase III (Worthington, LS004182). Splenocytes were isolated from Collagenase-treated spleens by density gradient centrifugation. Single-cell suspensions were stained, and lymphoid populations were characterized by flow cytometry as

described previously [6]. Briefly, all lymphocytes were characterized as $CD45^+$, T lymphocytes were $CD45^+$ $CD3^+$, natural killer (NK) cells were $CD45^+$ $Nk1.1^+$, Th cells were $CD3^+$ $CD4^+$ and CTL cells were $CD3^+$ $CD8^+$. The following antibodies were used: rat anti-mouse NK1.1-PE (Tonbo, 80-5941-U100, 1:100), anti-CD8-PE-cy7(Biolegend, 480035, 1:100), anti-CD45-APCCY7(BD, 550994, 1:100), anti-CD3-APC (Proteintech, 1:100) and anti-CD4-FITC (4A Bio, 1:100). Secondary antibodies, anti-rat Streptavidin-red 670 (Invitrogen, 1:1000), were used to conjugate the primary antibodies.

For tumor lymphocytes, all colon tumors from each mouse were pooled and washed three times in PBS. The tumors were minced in 5% FBS RPMI and enzymatically digested with 0.05% collagenase III for 30 minutes in a 37°C 5% CO_2 incubator. The digestion was stopped by adding DMEM complete medium, and the digested cells were passed through a 40 μM cell strainer to obtain single-cell suspensions. The cells were stained with anti-CD45, NK1.1, CD3, CD4 and CD8 antibodies. Fluorescent staining was carried out for 2 hours on ice at room temperature in the dark, followed by one wash with PBS. Flow cytometric analyses were conducted using Epics XL/MCL (Coulter, High Wycombe, United Kingdom) and dual-laser FACScan (Becton Dickinson) flow cytometers. FACSDiva (BD Bioscience) and FlowJo® software were utilized for cytometric analyses.

Statistical analysis

Statistical analysis was calculated using Prism software (GraphPad Prism 9). Unpaired two-tailed Student's *t*-test was employed to assess the statistical significance between the two groups. For multi-group comparisons, one-way ANOVA followed by Holm-Sidak's multiple comparisons test was conducted. Spearman correlation was used in the heatmap of intestinal microflora. Experiments were reproduced three times and pooled data are shown. $^*P < 0.05$, $^{**}P < 0.01$, $^{***}P < 0.001$, $^{****}P < 0.0001$, ns, no significance. Error Bars represent mean ± SEM.

Supplementary Table 1. The primers and probe used for quantitative real-time PCR reaction

Target	Primer (5'-3')	Product size (bp)	Primer Reference	FISH Probe (5'-3')	Probe Reference
UNI (16S)	F: ACTCCTACGGGAGGCAGCAGT		[8]		
	R: ATTACCGCGGCTGCTGGC				
C. jejuni hipO	F: AATGCACAAATTTGCCTTATAAAAGC	123	[26]	Cy3-AGCTAACCACACCTTATACCG	[9]
	R: TNCCATTAAAATTCTGACTTGCTAAATA				
ETBF (ATCC 43858)	F: AAGGGCTGGATGGCTTTACT	189	[2]	Cy3-GTTTCCACATCATTCCACTG	[5, 27] [28] [29] [30]
	R: GGGATACATCAGCTGGGTTG				
NTBF	F: CTCGGTATGGAGTATGCTCCAG	708	[5]		
	R: GTATTCCACTGCCCAATATGCCGC				
Bacteroides	F: GAGAGGAAGGTCCCCCAC		[31], [32]	BAC303(Alexa 514)-CCAATGTGGGGGACCTT	[31],[33]
	R: CGCTACTTGGCTGGTTCAG				
Bacteroidetes	F: GGARCATGTGGTTTAATTCGATGAT		[31], [34]		
	R: AGCTGACGACAACCATGCAG				

Supplementary Table 2. The list of genes significantly upregulated in the *C. jejuni* group compared to the mock group in the tumor tissue of *Apc*$^{min/+}$ mice.

Gene name	Full name	Fold change (*C. jejuni* vs Mock)	*P* value (*C. jejuni* vs Mock)	*P* value (Vaccine+*C. jejuni* vs mock)
Gm15104	predicted gene 15104	22.8	0.023132588	0.188440361
Tmprss12	transmembrane (C-terminal) protease, serine 12	9.58333333	0.003345489	0.902472719
Slc16a8	solute carrier family 16 (monocarboxylic acid transporters), member 8	7.07142857	0.021773042	0.086760412
Gm32234	predicted gene, 32234	6.32142857	0.038313938	0.042342053
Gnat3	guanine nucleotide binding protein, alpha transducing 3	5.83455882	0.046505833	0.483583783
T2	brachyury 2	5.67857143	0.026464499	0.121456334
Epha5	Eph receptor A5	5.54166667	0.013758688	0.466272903
Gm609	predicted gene 609	4.80894886	0.020982361	0.004093023
Ankrd63	ankyrin repeat domain 63	4.63888889	0.00714045	0.197790484
Apol7a	apolipoprotein L 7a	4.13579545	0.042540183	0.209686618
Slc17a1	solute carrier family 17 (sodium phosphate), member 1	3.87857143	0.00996021	0.218247436
Tat	tyrosine aminotransferase	3.37665198	0.009588863	0.397321883

Clca3b	chloride channel accessory 3B	3.3133264	0.028264611	0.286378533
Nox1	NADPH oxidase 1	3.04038462	0.048406444	0.087813504
Ms4a5	membrane-spanning 4-domains, subfamily A, member 5	2.84693878	0.035409316	0.388911765
Hmga2	high mobility group AT-hook 2	2.80796586	0.043009829	0.333620234
Gng13	guanine nucleotide binding protein (G protein), gamma 13	2.65819672	0.033262801	0.023634085
Ism2	isthmin 2	2.625	0.037281601	0.363746112
Spats2l	spermatogenesis associated, serine-rich 2-like	2.51923817	0.009786844	0.131081964
Hk2	hexokinase 2	2.46832011	0.018141204	0.103451122
Map3k13	mitogen-activated protein kinase kinase kinase 13	2.37478992	0.042516088	0.052064347
Cdcp1	CUB domain containing protein 1	2.32668303	0.047198731	0.069488233
Dusp8	dual specificity phosphatase 8	2.28111495	0.028530811	0.13204494
Trib1	tribbles pseudokinase 1	2.18365466	0.040355472	0.081335448
Grip1	glutamate receptor interacting protein 1	2.14795918	0.044692926	0.521634241
Ppp1r14c	protein phosphatase 1, regulatory inhibitor subunit 14C	2.1122449	0.044166583	0.141633601

Supplementary Table 3. The list of genes significantly downregulated in the *C. jejuni* group compared to the mock group in the tumor tissue of *Apc*$^{min/+}$ mice.

Gene name	Full name	Fold change (*C. jejuni* vs Mock)	*P* value (*C. jejuni* vs Mock)	*P* value (Vaccine+*C. jejuni* vs mock)
Prss50	protease, serine 50	0	0.00950197	0.97574699
Meig1	meiosis expressed gene 1	0	0.00625089	0.22121096
Gm7160	predicted gene 7160	0	0.0313962	0.13819673
B230311B06Rik	RIKEN cDNA B230311B06 gene	0	0.0036093	0.05412378
Kcnk12	potassium channel, subfamily K, member 12	0	0.00047209	0.03742265
Slco1a4	solute carrier organic anion transporter family, member 1a4	0	0.01158918	0.01158918
Gm5544	predicted gene 5544	0	4.7143E-07	4.7143E-07
Drd1	dopamine receptor D1	0.09677419	0.0416032	0.02365413
Gm38958	predicted gene, 38958	0.11538462	0.02562831	0.30229092
Ccl27b	chemokine (C-C motif) ligand 27b	0.11619718	0.01678899	0.00895167
Rnase10	ribonuclease, RNase A family, 10 (non-active)	0.12272727	0.02497016	0.04284691
Vmn1r90	vomeronasal 1 receptor 90	0.14	0.01547378	0.43894184
Gm10705	predicted gene 10705	0.15425532	0.01679419	0.19328914
Nxph4	neurexophilin 4	0.15753425	0.04694311	0.05094155
Gm14853	predicted gene 14853	0.17213115	0.0466436	0.16635794
Crlf1	cytokine receptor-like factor 1	0.17404383	0.04191039	0.07988144
Mucl3	mucin like 3	0.18	0.04883913	0.33252054
Kbtbd12	kelch repeat and BTB (POZ) domain containing 12	0.18157895	0.02182689	0.02140573
Ccdc194	coiled-coil domain containing 194	0.1875	0.04462924	0.04462924
Gm46345	predicted gene, 46345	0.1875	0.04462924	0.00496662
LOC100861978	c-C motif chemokine 27-like	0.19542254	0.02383294	0.00841131

Cldn19	claudin 19	0.20454545	0.03877851	0.03877851
Pnma5	paraneoplastic antigen family 5	0.20833333	0.02833075	5.9951E-05
Dbp	D site albumin promoter binding protein	0.2127907	0.04094282	0.37549574
Daw1	dynein assembly factor with WDR repeat domains 1	0.21428571	0.02754466	0.54040295
Hist1h4a	histone cluster 1, H4a	0.23235294	0.0047852	0.01506448
Gm3264	predicted gene 3264	0.23460591	0.03393667	0.05220947
Gm13306	predicted gene 13306	0.24152542	0.01213764	0.00106655
Xlr3b	X-linked lymphocyte-regulated 3B	0.24810791	0.01253123	0.94675145
Hist1h2af	histone cluster 1, H2af	0.25131926	0.02302215	0.13584518
Slc16a4	solute carrier family 16 (monocarboxylic acid transporters), member 4	0.26298701	0.03460771	0.05310631
D1Pas1	DNA segment, Chr 1, Pasteur Institute 1	0.29166667	0.03485074	0.05470711
Tmsb15b1	thymosin beta 15b1	0.29802053	0.01778077	0.21825046
Mtnr1a	melatonin receptor 1A	0.30357143	0.04215352	0.83860044
Gm17455	predicted gene, 17455	0.30833333	0.04074836	0.30157239
Aipl1	aryl hydrocarbon receptor-interacting protein-like 1	0.32038835	0.00969874	0.1046975
Gm43305	predicted gene 43305	0.32312893	0.00729096	0.42896181
Dppa3	developmental pluripotency-associated 3	0.33266129	0.01327525	0.44934399
Adprhl1	ADP-ribosylhydrolase like 1	0.34821429	0.03638302	0.22352919
B3galt1	UDP-Gal:betaGlcNAc beta 1,3-galactosyltransferase, polypeptide 1	0.35526316	0.01815832	0.02645855
Lipm	lipase, family member M	0.35526316	0.03186612	0.11975464
Gm29485	predicted gene 29485	0.37820513	0.00497741	0.08538155
Prcd	photoreceptor disc component	0.37909091	0.03165881	0.92699459
Pcdhga2	protocadherin gamma subfamily A, 2	0.40223464	0.00419614	0.03016257
4931423N10Rik	RIKEN cDNA 4931423N10 gene	0.40625	0.0319523	0.14381081
Gm39743	predicted gene, 39743	0.42375307	0.010024	0.47025606
Nupr1l	nuclear protein transcriptional regulator 1 like	0.42915905	0.04465372	0.64579206
Lrrn2	leucine rich repeat protein 2, neuronal	0.43246562	0.03899138	0.51608654
Smim4	small integral membrane protein 4	0.43286201	0.02166058	0.08936479
Gm41476	predicted gene, 41476	0.44413636	0.01368963	0.55965173
Omd	osteomodulin	0.44890511	0.01688436	0.02977332
Ccdc62	coiled-coil domain containing 62	0.45538922	0.03196711	0.16176458
Spag8	sperm associated antigen 8	0.462	0.01493277	0.89117424
Padi2	peptidyl arginine deiminase, type II	0.46863905	0.02212919	0.81587095
Rsph1	radial spoke head 1 homolog (Chlamydomonas)	0.48840942	0.03998399	0.18169109

Supplementary Table 4. The list of genes significantly upregulated in the *C. jejuni* group compared to the mock group in the para-cancerous tissue of *Apc*$^{min/+}$ mice.

Gene name	Full name	Fold change (*C. jejuni* vs Mock)	*P* value (*C. jejuni* vs Mock)	*P* value (Vaccine+*C. jejuni* vs mock)
Erv3	endogenous retroviral sequence 3	8.5	0.00237536	0.18207385
Lrrc34	leucine rich repeat containing 34	7.5	0.01662821	0.04967366
Rpl3l	ribosomal protein L3-like	7	0.00320148	0.15553038
Spem1	sperm maturation 1	6.75	0.03063917	0.61744803
Gm2808	predicted gene 2808	6.65625	0.01127106	0.2895408
Gm33933	predicted gene, 33933	6.5625	0.00265059	0.38741157
Peg12	paternally expressed 12	5.8125	0.01210685	0.00052521
Rgsl1	regulator of G-protein signaling like 1	5.75	0.00815815	0.04565912
Acsm5	acyl-CoA synthetase medium-chain family member 5	5.25	0.02250728	0.41219078
Slc16a14	solute carrier family 16 (monocarboxylic acid transporters), member 14	5	0.03271994	0.19351755
Trhde	TRH-degrading enzyme	4.375	0.03981235	0.23677068
Phkg1	phosphorylase kinase gamma 1	4.0078125	0.01916679	0.10785203
Pnldc1	poly(A)-specific ribonuclease (PARN)-like domain containing 1	4	0.00455399	0.46711529
3830403N18Rik	RIKEN cDNA 3830403N18 gene	3.84016393	0.04163511	0.24582045
Dnah7c	dynein, axonemal, heavy chain 7C	3.75	0.03785673	0.3524132
Dppa3	developmental pluripotency-associated 3	3.725	0.04497994	0.50947569
Csrnp3	cysteine-serine-rich nuclear protein 3	3.64285714	0.04426154	0.01551898
Mnx1	motor neuron and pancreas homeobox 1	3.60766423	0.00785388	0.93506637
Ankrd61	ankyrin repeat domain 61	3.47014925	0.01077084	0.22067166
Pnpla3	patatin-like phospholipase domain containing 3	3.42692308	0.00505339	0.48933879
Tshr	thyroid stimulating hormone receptor	3.34821429	0.00874983	0.96479665
Pvrig	poliovirus receptor related immunoglobulin domain containing	3.24519231	0.01093855	0.43107562
Klre1	killer cell lectin-like receptor family E member 1	3.21153846	0.03848556	0.15729632
Sirpb1b	signal-regulatory protein beta 1B	3.20121951	0.00085277	0.00066624
Fam228b	family with sequence similarity 228, member B	3.07317073	0.04980108	0.04167801
Fgf2	fibroblast growth factor 2	2.93965517	0.04784953	0.37962984
Gm2102	predicted gene 2102	2.8828125	0.04441598	0.16016371
Ptgs2	prostaglandin-endoperoxide synthase 2	2.88230769	0.04327603	0.05751062
Grip1	glutamate receptor interacting protein 1	2.88109756	0.04436632	0.00032779
Gpr62	G protein-coupled receptor 62	2.86363636	0.04374671	0.31756587
Trpa1	transient receptor potential cation channel, subfamily A, member 1	2.86280488	0.02964548	0.00996151
Gcnt7	glucosaminyl (N-acetyl) transferase family member 7	2.83695652	0.03134719	0.88346562
Myl1	myosin, light polypeptide 1	2.73489426	0.01525266	0.61917153

Adrb3	adrenergic receptor, beta 3	2.70258621	0.04924345	0.76053218
LOC102639021	zinc finger protein 431-like	2.625	0.01590016	0.29917961
Rbm11	RNA binding motif protein 11	2.59756098	0.00639239	0.10529992
Thrsp	thyroid hormone responsive	2.55731707	0.03274551	0.78490208
Rasgrf1	RAS protein-specific guanine nucleotide-releasing factor 1	2.51785714	0.01757071	0.06539891
Opcml	opioid binding protein/cell adhesion molecule-like	2.51351351	0.02153372	0.0138384
Csgalnact1	chondroitin sulfate N-acetylgalactosaminyltransferase 1	2.5125	0.01723887	0.00060159
Alkal2	ALK and LTK ligand 2	2.48076923	0.00817634	0.17462286
Gm46310	predicted gene, 46310	2.46982759	0.01142679	0.26590698
Tbx6	T-box 6	2.46014493	0.04129633	0.21046221
Lrrc18	leucine rich repeat containing 18	2.39516129	0.01817951	0.02212828
Kcng3	potassium voltage-gated channel, subfamily G, member 3	2.39285714	0.01190658	0.11962924
Pld5	phospholipase D family, member 5	2.34246575	0.02141755	0.15081587
Mroh6	maestro heat-like repeat family member 6	2.33690987	0.01426869	0.0930544
Cldn34c1	claudin 34C1	2.33478261	0.02601994	0.00494334
Areg	amphiregulin	2.28691783	0.03391921	0.52619073
Dclk3	doublecortin-like kinase 3	2.26576577	0.04852565	0.0031208
Ear2	eosinophil-associated, ribonuclease A family, member 2	2.22421203	0.04533078	0.49481369
Casq2	calsequestrin 2	2.20369433	0.02067895	0.0074749
Ncam2	neural cell adhesion molecule 2	2.1875	0.03181628	0.07600537
Il12rb2	interleukin 12 receptor, beta 2	2.12903226	0.03793338	0.04946008
Mamdc4	MAM domain containing 4	2.12790698	0.02942409	0.08631392
Gria4	glutamate receptor, ionotropic, AMPA4 (alpha 4)	2.11702128	0.03645065	0.03681223
Fabp7	fatty acid binding protein 7, brain	2.1	0.04876106	0.27861394
Lsmem2	leucine-rich single-pass membrane protein 2	2.08928571	0.01808373	0.02538847
Lmo3	LIM domain only 3	2.07272727	0.01518294	0.0351966
Mfsd4b5	major facilitator superfamily domain containing 4B5	2.07178218	0.00740116	0.02874587
Zfp286	zinc finger protein 286	2.06686047	0.03060208	0.0325789
Trpc1	transient receptor potential cation channel, subfamily C, member 1	2.06052632	0.04104363	0.01131414

Supplementary Table 5. The list of genes significantly downregulated in the *C. jejuni* group compared to the mock group in the para-cancerous tissue of *Apc*$^{min/+}$ mice.

Gene name	Full name	Fold change (*C. jejuni* vs Mock)	*P* value (*C. jejuni* vs Mock)	*P* value (Vaccine+*C. jejuni* vs mock)
Gjd3	gap junction protein, delta 3	0	0.00010488	0.10280009
Hist1h1t	histone cluster 1, H1t	0	0.00035119	0.06171242
LOC630751	interferon-inducible GTPase 1-like	0	0.00977597	0.81318534
Lexm	lymphocyte expansion molecule	0	0.0108199	0.0402846
Smc1b	structural maintenance of chromosomes 1B	0	0.01480293	0.01480293
Gm40824	predicted gene, 40824	0	0.04809055	0.78179587
Dsg1c	desmoglein 1 gamma	0.07894737	0.00760836	0.63473353
Myadml2	myeloid-associated differentiation marker-like 2	0.10294118	0.01002297	0.10823413
Fam205a3	family with sequence similarity 205, member A3	0.10714286	0.03662812	0.61155193
Hist1h2ah	histone cluster 1, H2ah	0.16546763	0.04381881	0.12006903
Vmn1r181	vomeronasal 1 receptor 181	0.17045455	0.01731267	0.00048719
Adam32	a disintegrin and metallopeptidase domain 32	0.1875	0.0363815	0.56411357
Epha10	Eph receptor A10	0.1875	0.04462924	0.08185445
Ptprq	protein tyrosine phosphatase, receptor type, Q	0.1875	0.04462924	0.04462924
Poln	DNA polymerase N	0.21428571	0.03785673	0.95952229
Fhl4	four and a half LIM domains 4	0.22297297	0.03737469	0.05050834
Cyp26a1	cytochrome P450, family 26, subfamily a, polypeptide 1	0.23275862	0.04789063	0.59879995
Gm3417	predicted gene 3417	0.23489011	0.0348744	0.34770196
Spaca7	sperm acrosome associated 7	0.24652778	0.00238831	0.00073754
Ucn3	urocortin 3	0.253125	0.01611632	0.48548485
Ces3a	carboxylesterase 3A	0.26326531	0.04725286	0.13883007
Prss33	protease, serine 33	0.27272727	0.04531877	0.06630055
Hist2h3c2	histone cluster 2, H3c2	0.27773775	0.01315979	0.05561778
Npffr1	neuropeptide FF receptor 1	0.29347826	0.03597796	0.84364416
Hist2h3b	histone cluster 2, H3b	0.32643885	0.0424644	0.19583618
St6galnac1	ST6 (alpha-N-acetyl-neuraminyl-2,3-beta-galactosyl-1,3)-N-acetylgalactosaminide alpha-2,6-sialyltransferase 1	0.38038674	0.01717749	0.00769454
Slpi	secretory leukocyte peptidase inhibitor	0.39355999	0.03693337	0.0940877
Epha8	Eph receptor A8	0.4	0.03149041	0.20397389
Cd177	CD177 antigen	0.40106248	0.03392658	0.07780437
Klk10	kallikrein related-peptidase 10	0.40178571	0.04338535	0.0980749
1110032F04Rik	RIKEN cDNA 1110032F04 gene	0.41752577	0.02946551	0.48298769
Ccdc190	coiled-coil domain containing 190	0.44620253	0.04958948	0.145516
Cpz	carboxypeptidase Z	0.46195652	0.03715569	0.41287787
Gm20684	predicted gene 20684	0.47635135	0.00354259	0.32168092

Supplementary References

1. P. K. Talukdar, N. M. Negretti, K. L. Turner, M. E. Konkel, Molecular Dissection of the Campylobacter jejuni CadF and FlpA Virulence Proteins in Binding to Host Cell Fibronectin. *Microorganisms* **8** (2020).
2. A. L. Hecht *et al.*, Strain competition restricts colonization of an enteric pathogen and prevents colitis. *EMBO Rep* **17**, 1281-1291 (2016).
3. N. Giallourou *et al.*, A novel mouse model of Campylobacter jejuni enteropathy and diarrhea. *PLoS Pathog* **14**, e1007083 (2018).
4. H. Chen *et al.*, Antibiotic-induced microbiome depletion promotes intestinal colonization by Campylobacter jejuni in mice. *BMC Microbiol* **24**, 156 (2024).
5. J. L. Chan *et al.*, Non-toxigenic Bacteroides fragilis (NTBF) administration reduces bacteria-driven chronic colitis and tumor development independent of polysaccharide A. *Mucosal Immunol* **12**, 164-177 (2019).
6. S. Wu *et al.*, A human colonic commensal promotes colon tumorigenesis via activation of T helper type 17 T cell responses. *Nat Med* **15**, 1016-1022 (2009).
7. W. Zheng *et al.*, Microbiota-targeted maternal antibodies protect neonates from enteric infection. *Nature* **577**, 543-548 (2020).
8. E. Zagato *et al.*, Endogenous murine microbiota member Faecalibaculum rodentium and its human homologue protect from intestinal tumour growth. *Nat Microbiol* **5**, 511-524 (2020).
9. X. Sun, D. Threadgill, C. Jobin, Campylobacter jejuni induces colitis through activation of mammalian target of rapamycin signaling. *Gastroenterology* **142**, 86-95.e85 (2012).
10. K. J. Rhee *et al.*, Induction of persistent colitis by a human commensal, enterotoxigenic Bacteroides fragilis, in wild-type C57BL/6 mice. *Infect Immun* **77**, 1708-1718 (2009).
11. G. P. Boivin *et al.*, Pathology of mouse models of intestinal cancer: consensus report and recommendations. *Gastroenterology* **124**, 762-777 (2003).
12. W. Zhu *et al.*, Editing of the gut microbiota reduces carcinogenesis in mouse models of colitis-associated colorectal cancer. *J Exp Med* **216**, 2378-2393 (2019).
13. X. Sun *et al.*, Microbiota-Derived Metabolic Factors Reduce Campylobacteriosis in Mice. *Gastroenterology* **154**, 1751-1763.e1752 (2018).
14. A. M. Schmidt *et al.*, Immunopathological properties of the Campylobacter jejuni flagellins and the adhesin CadF as assessed in a clinical murine infection model. *Gut Pathog* **11**, 24 (2019).
15. T. Bo *et al.*, Effects of High-Fat Diet During Childhood on Precocious Puberty and Gut Microbiota in Mice. *Front Microbiol* **13**, 930747 (2022).
16. E. Bolyen *et al.*, Reproducible, interactive, scalable and extensible microbiome data science using QIIME 2. *Nat Biotechnol* **37**, 852-857 (2019).
17. B. Wen, Z. Mei, C. Zeng, S. Liu, metaX: a flexible and comprehensive software for processing metabolomics data. *BMC Bioinformatics* **18**, 183 (2017).
18. A. M. Bolger, M. Lohse, B. Usadel, Trimmomatic: a flexible trimmer for Illumina sequence data. *Bioinformatics* **30**, 2114-2120 (2014).
19. B. Langmead, S. L. Salzberg, Fast gapped-read alignment with Bowtie 2. *Nat Methods* **9**, 357-359 (2012).
20. C. Trapnell *et al.*, Differential gene and transcript expression analysis of RNA-seq experiments with TopHat and Cufflinks. *Nat Protoc* **7**, 562-578 (2012).

21. Y. Liao, G. K. Smyth, W. Shi, featureCounts: an efficient general purpose program for assigning sequence reads to genomic features. *Bioinformatics* **30**, 923-930 (2014).

22. M. I. Love, W. Huber, S. Anders, Moderated estimation of fold change and dispersion for RNA-seq data with DESeq2. *Genome Biol* **15**, 550 (2014).

23. M. Kanehisa, S. Goto, KEGG: kyoto encyclopedia of genes and genomes. *Nucleic Acids Res* **28**, 27-30 (2000).

24. A. Blanco-Miguez *et al.*, Extending and improving metagenomic taxonomic profiling with uncharacterized species using MetaPhlAn 4. *Nat Biotechnol* 10.1038/s41587-023-01688-w (2023).

25. F. Beghini *et al.*, Integrating taxonomic, functional, and strain-level profiling of diverse microbial communities with bioBakery 3. *Elife* **10** (2021).

26. A. Sarabi Asiabar *et al.*, Molecular detection of Campylobacter jejuni in patients with Crohn's disease in Iran. *Med J Islam Repub Iran* **33**, 76 (2019).

27. C. M. Dejea *et al.*, Microbiota organization is a distinct feature of proximal colorectal cancers. *Proc Natl Acad Sci U S A* **111**, 18321-18326 (2014).

28. C. M. Dejea *et al.*, Patients with familial adenomatous polyposis harbor colonic biofilms containing tumorigenic bacteria. *Science* **359**, 592-597 (2018).

29. S. Guo *et al.*, Downregulation of the farnesoid X receptor promotes colorectal tumorigenesis by facilitating enterotoxigenic Bacteroides fragilis colonization. *Pharmacol Res* **177**, 106101 (2022).

30. A. Saffarian *et al.*, Crypt- and Mucosa-Associated Core Microbiotas in Humans and Their Alteration in Colon Cancer Patients. *mBio* **10** (2019).

31. T. Pédron *et al.*, A crypt-specific core microbiota resides in the mouse colon. *mBio* **3** (2012).

32. X. Guo *et al.*, Development of a real-time PCR method for Firmicutes and Bacteroidetes in faeces and its application to quantify intestinal population of obese and lean pigs. *Lett Appl Microbiol* **47**, 367-373 (2008).

33. W. Manz, R. Amann, W. Ludwig, M. Vancanneyt, K. H. Schleifer, Application of a suite of 16S rRNA-specific oligonucleotide probes designed to investigate bacteria of the phylum cytophaga-flavobacter-bacteroides in the natural environment. *Microbiology (Reading)* **142 (Pt 5)**, 1097-1106 (1996).

34. A. Rehman *et al.*, Nod2 is essential for temporal development of intestinal microbial communities. *Gut* **60**, 1354-1362 (2011).

SUPPLEMENTARY FIGURES AND FIGURE LEGENDS

Supplementary Figure 1

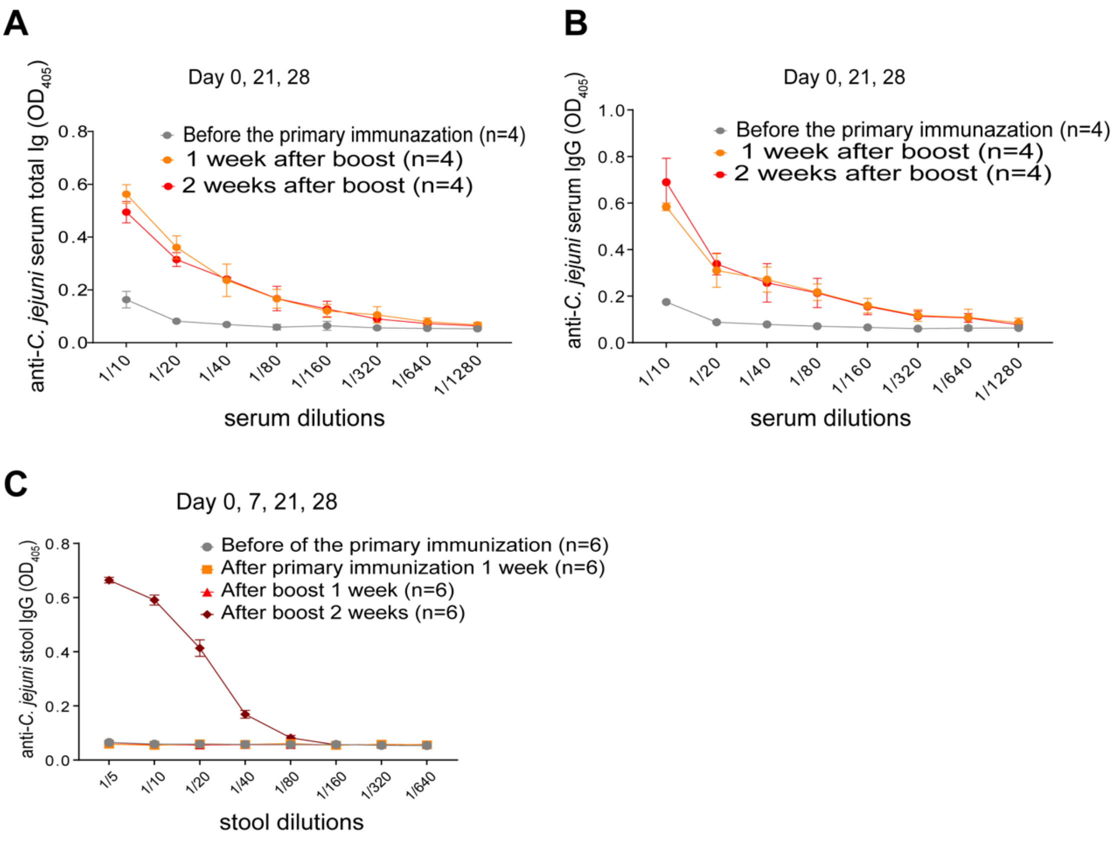

Supplementary Figure 1: The *C. jejuni* vaccine elicits anti-*C. jejuni* antibodies in vaccine-*C. jejuni* mice at various time points. (A-B) Total immunoglobulin, and IgG titers against *C. jejuni* in serum at different time points (Day 0, 21, 28) from Vaccine-*C. jejuni* mice. (C) IgG titers against *C. jejuni* in stool from Vaccine-*C. jejuni* mice in the different time points (Day 0, 7, 21, 28). Data are mean ± SEM. Specific n numbers are indicated in the figure. Data are representative of two independent experiments. OD_{405}, optical density at 405 nm.

Supplementary Figure 2

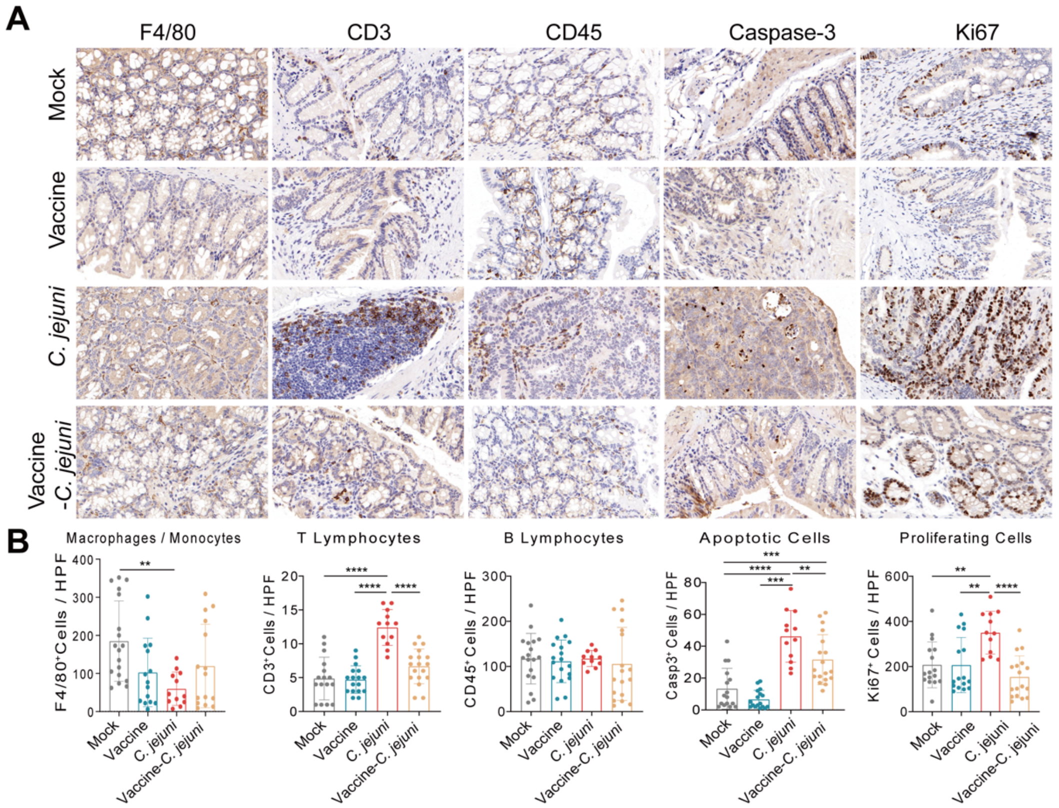

Supplementary Figure 2: The effect of the *C. jejuni* vaccine and *C. jejuni* infection on lymphocyte and cell apoptosis and proliferation in the preventive model. (A) Representative IHC images of T lymphocytes (CD3+), B lymphocytes (CD45+), colonic epithelial apoptotic cells (positive for caspase-3, Casp3), macrophages and monocytes (F4/80+) and proliferating/regenerating cells (positive for Ki67) staining in mice. Scale bar, 20μm. (B) Percentage of T lymphocytes (CD3+), B lymphocytes (CD45+), colonic epithelial apoptotic cells (positive for caspase-3, Casp3), macrophages and monocytes (F4/80+) and proliferating/regenerating cells (positive for Ki67) from mock, vaccine, *C. jejuni* and Vaccine-*C. jejuni* mice; n = 3 mice/group. Dot blots show corresponding quantitative analysis of CD3, CD45, Caspase-3, F4/80, and Ki67 staining. Each dot represents a high power field (HPF). Data from two independent experiments are represented as the mean ± s.e.m. Specific n numbers are indicated in the figure. *P* values were calculated by one-way ANOVA with Holm–Sidak for multiple comparisons, **P* < 0.05, ***P* < 0.01, ****P* < 0.001, *****P* < 0.0001, ns, no significance. Error Bars represent mean ± SEM.

Supplementary Figure 3

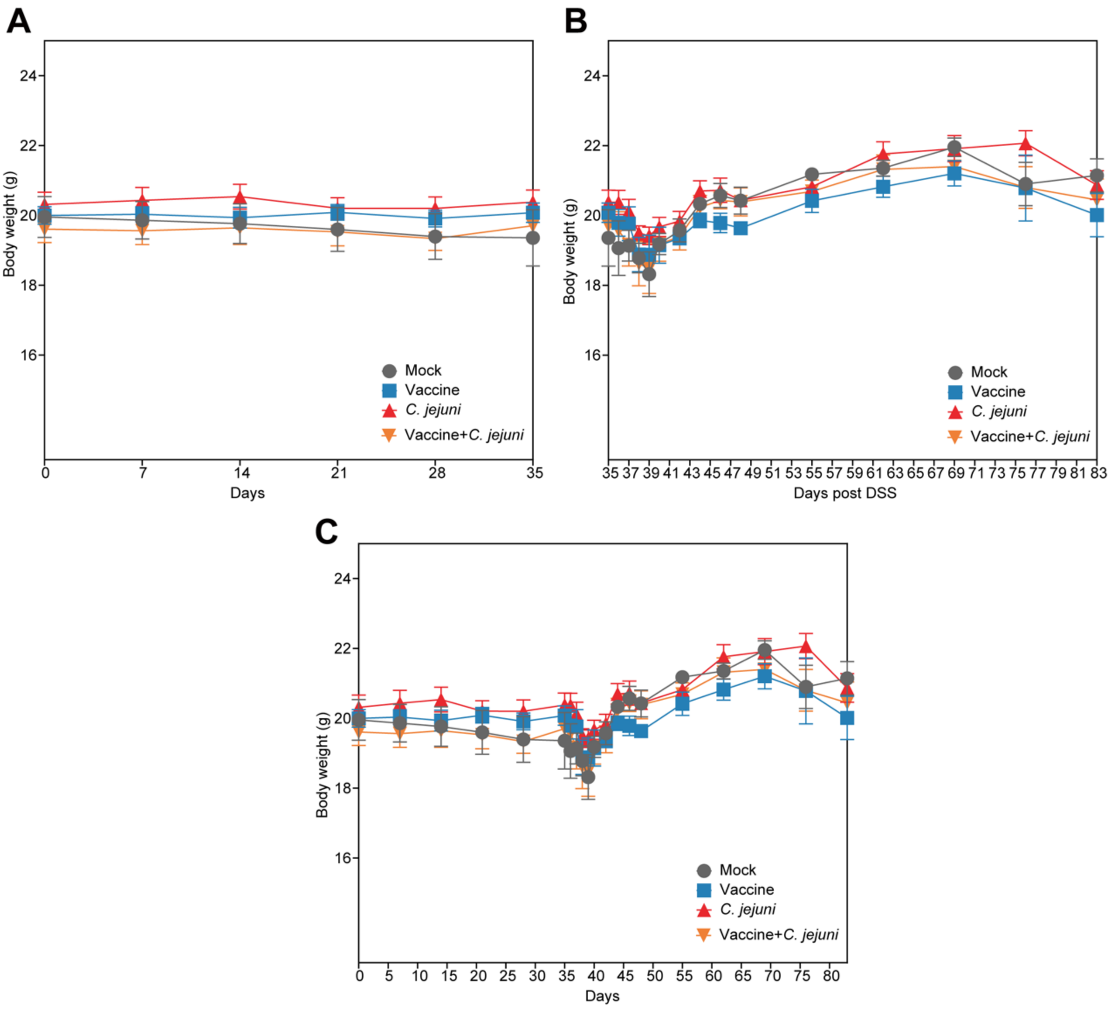

Supplementary Figure 3: The *C. jejuni* vaccine does not affect the body weight of mice. (A) Body weights of mice from day 0 to day 35 during the experiments. (B) Body weights of mice from day 35 to day 83 during the experiments. (C) Body weights of mice from day 0 to day 83 during the experiments. Data are representative of two independent experiments. On day 0 and day 14, the mice in the vaccine group and the vaccine + C. jejuni group were administered subcutaneous injections of the vaccine. From day 35 to day 40, all mice were given DSS water treatment.

Supplementary Figure 4

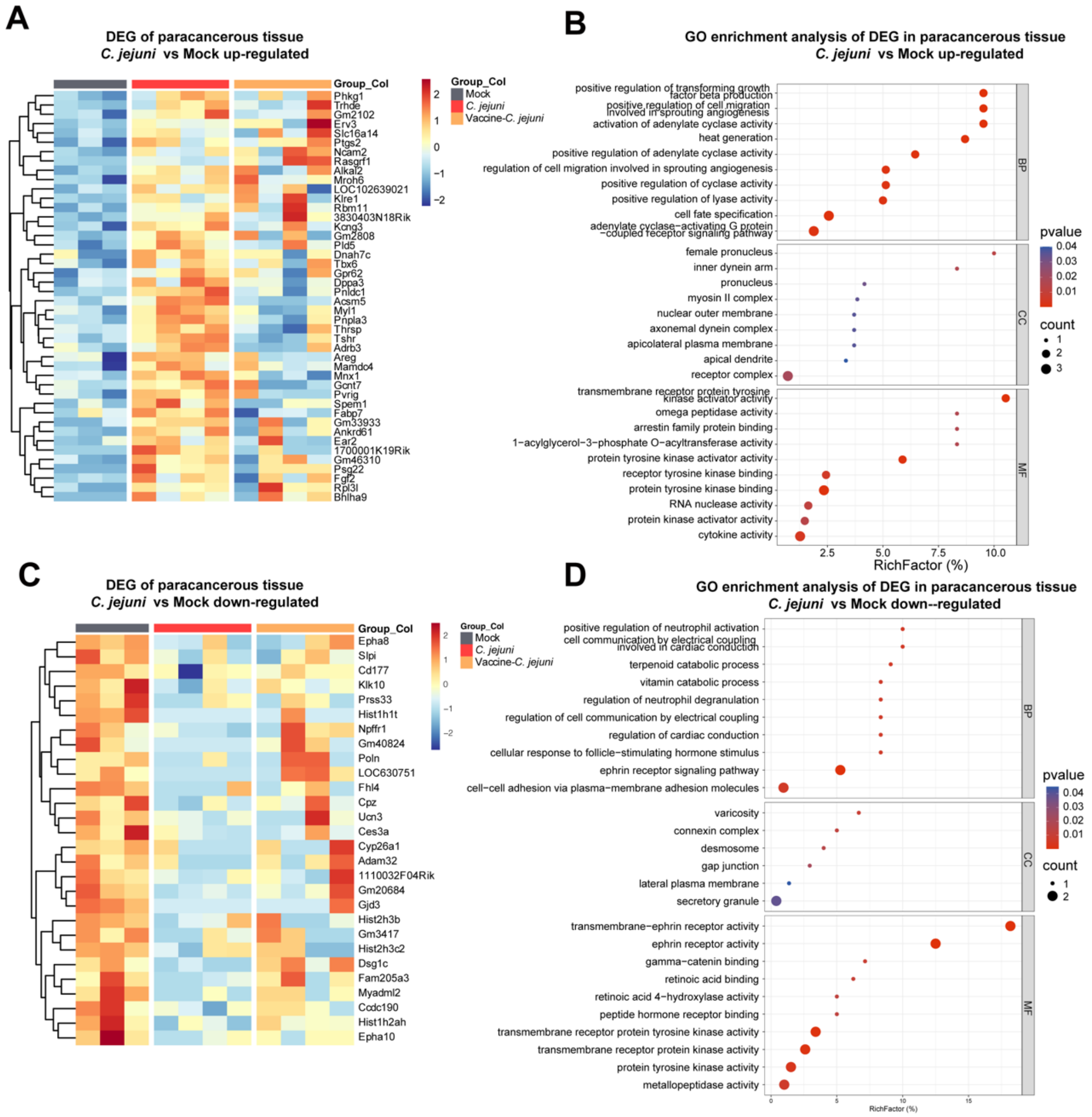

Supplementary Figure 4: Effect of inactivated vaccine on differentially expressed genes from the paracancerous tissue of the colon in *Apc*$^{min/+}$ DSS CRC mice. RNA-seq was performed on RNA from paracancerous tissues in the colon of mice from mock (n = 3), *C. jejuni* (n = 4), and Vaccine-*C. jejuni* group (n = 4). (A) Clustered heatmap of significantly up-regulated expressed genes in *C. jejuni* group compared to the mock group, whereas no significant difference between the mock group and the vaccine-*C. jejuni* group. The bar color for each sample indicates the treatment, mock (grey), *C. jejuni* (red), or Vaccine-*C. jejuni* (orange). (B) GO enrichment analysis of up-regulated genes in paracancerous tissues of mouse colon in the *C. jejuni* group compared to the mock group. BP: Biological process; CC: Cellular component; MF: Molecular function. (C) Clustered

heatmap of significantly down-regulated expressed genes in *C. jejuni* group compared to the mock group, whereas no significant difference between the mock group and the vaccine-*C. jejuni* group. The bar color for each sample indicates the treatment, mock (grey), *C. jejuni* (red), or Vaccine-*C. jejuni* (orange). (D) GO enrichment analysis of down-regulated genes in paracancerous tissues of mouse colon in the *C. jejuni* group compared to the mock group. BP: Biological process; CC: Cellular component; MF: Molecular function.

Supplementary Figure 5

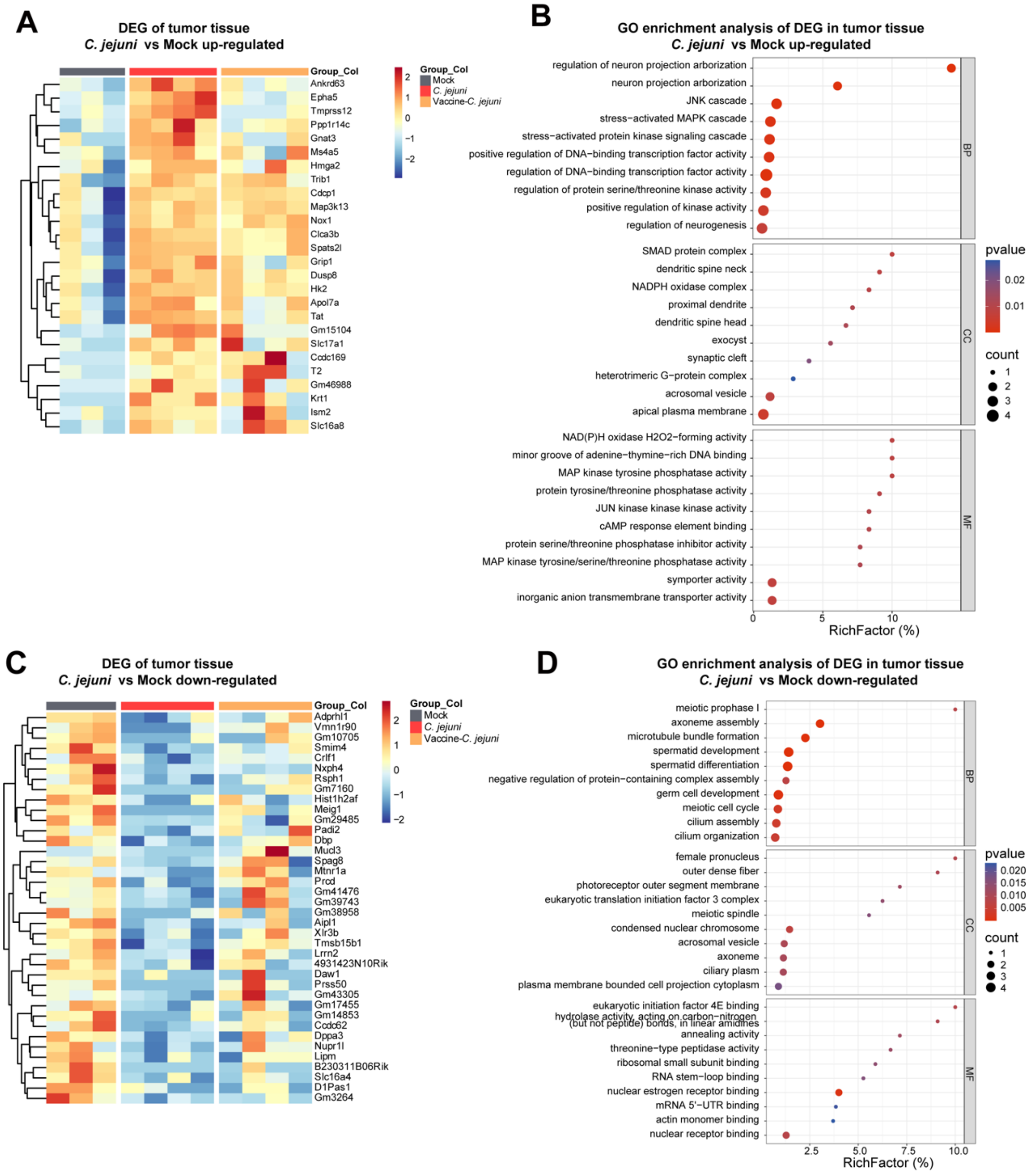

Supplementary Figure 5: Effect of inactivated *C. jejuni* vaccine on gene expression within the tumor tissue of the colon in *Apc*$^{min/+}$ DSS CRC mice. RNA-seq was performed on RNA from tumor tissues in the colon of mice from mock (n = 3), *C. jejuni* (n = 4), and vaccine-*C. jejuni* group (n = 4). (A) Clustered heatmap of significantly up-regulated expressed genes in the *C. jejuni* group compared to the mock group, whereas no significant difference between the mock group and the vaccine-*C. jejuni* group. Dendrograms cluster genes based on gene expression profiling similarity. The bar color for each sample indicates the treatment, mock (grey), *C. jejuni* (red), or Vaccine-*C. jejuni*

(orange). (B) GO enrichment analysis of up-regulated genes in tumor tissues of mouse colon in the *C. jejuni* group compared to the mock group. BP: Biological process; CC: Cellular component; MF: Molecular function. (C) Clustered heatmap of significantly down-regulated expressed genes in the *C. jejuni* group compared to the mock group, whereas no significant difference between the mock group and the vaccine-*C. jejuni* group. Dendrograms cluster genes based on gene expression profiling similarity. The bar color for each sample indicates the treatment, mock (grey), *C. jejuni* (red), or Vaccine-*C. jejuni* (orange). (D) GO enrichment analysis of down-regulated genes in tumor tissues of mouse colon in the *C. jejuni* group compared to the mock group. BP: Biological process; CC: Cellular component; MF: Molecular function.

Supplementary Figure 6

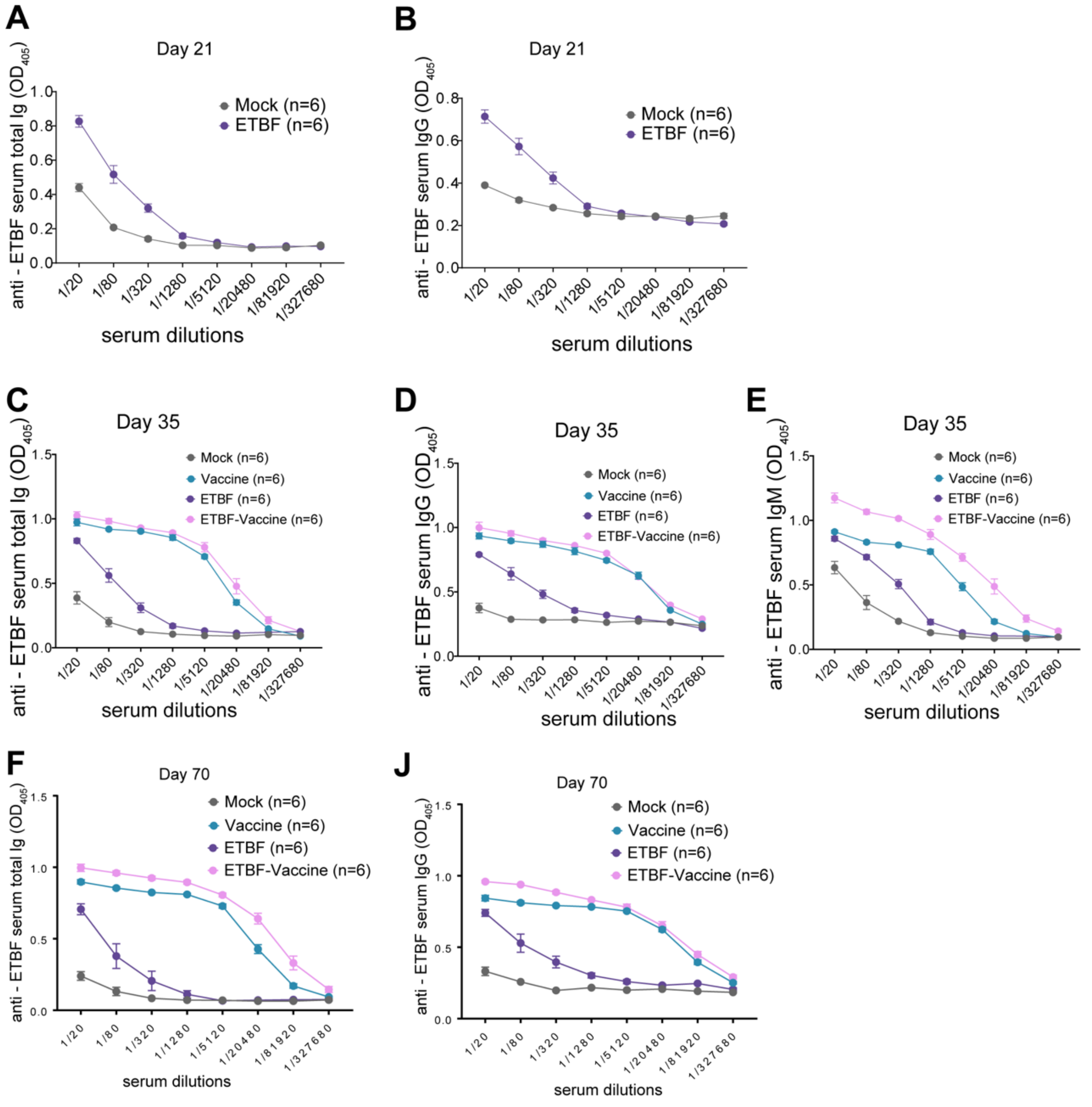

Supplementary Figure 6: The ETBF vaccine elicits anti-ETBF sera antibodies in mice serum following ETBF infection and vaccination. (A-B) Total and IgG immunoglobulin titers against ETBF in serum after infection 2 weeks (Day 21) from mock and ETBF mice. (C-E) Total, IgG, and IgM immunoglobulin titers against ETBF in serum after primary immunity 2 weeks (day 35) from the mock, vaccine, ETBF, and Vaccine-ETBF mice. (F-J) Total and IgG immunoglobulin titers against ETBF in serum at endpoint (day 70) from mock, vaccine, ETBF, and vaccine-ETBF mice. Data are representative of two independent experiments. OD_{405}, optical density at 405 nm. Data are mean ± SEM. Specific n numbers are indicated in the figure.

Supplementary Figure 7

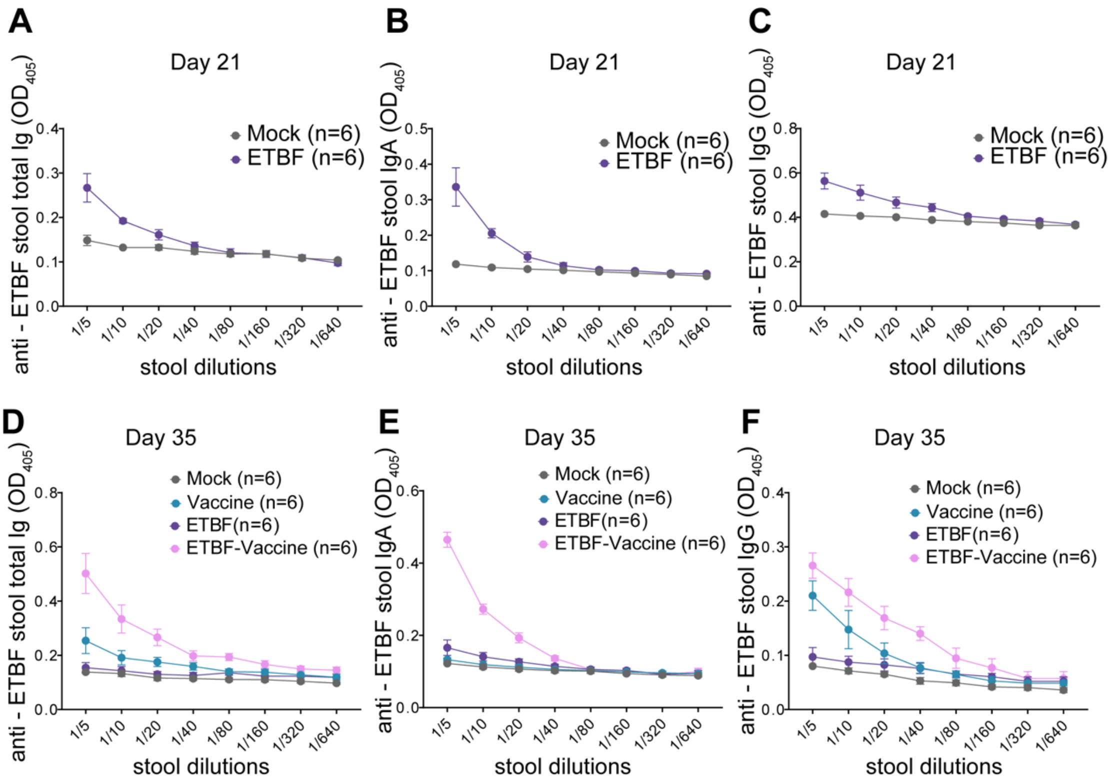

Supplementary Figure 7: The ETBF vaccine elicits anti-ETBF antibodies in mice feces following ETBF infection and vaccination. (A-C) Total, IgA, and IgG immunoglobulin titers against ETBF in stool after infection 2 weeks (Day 21) from mock and ETBF mice. (E-F) Total, IgA, and IgG immunoglobulin titers against ETBF in stool after primary immunity 2 weeks (day 35) from mock, vaccine, ETBF, and Vaccine-ETBF mice. OD_{405}, optical density at 405 nm. Data are mean ± SEM. Specific n numbers are indicated in the figure.

Supplementary Figure 8

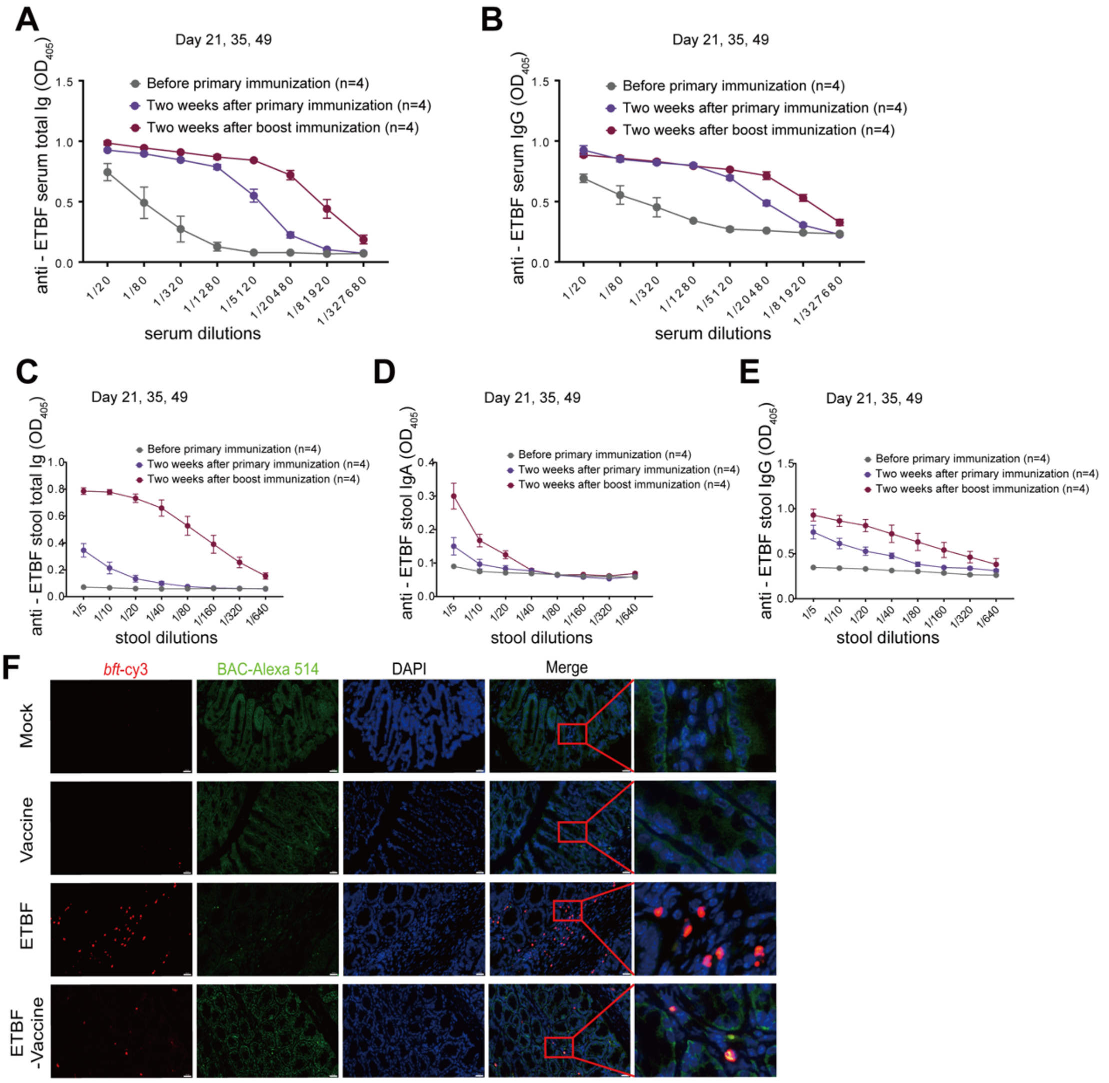

Supplementary Figure 8: **The ETBF vaccine elicits anti-ETBF antibodies following ETBF infection and vaccination and effectively reduces the oncomicrobes burden.** (A and B) Total and IgG immunoglobulin titers against ETBF in serum at different time points (day 21, 35, 49) from ETBF-Vaccine mice. (C- E) Total, IgA, and IgG immunoglobulin titers in serum at different time points (day 21, 35, 49) from ETBF-Vaccine mice. Data are representative of two independent experiments. OD_{405}, optical density at 405 nm. Error Bars represent mean ± SEM. Specific n numbers are indicated in the figure. (F) FISH using a Cy3-labeled ETBF-specific probe (red) and Alexa 514-labeled Bacteroides-specific probe (green) on colonic sections from the Swiss-rolled colon at the endpoint. Nuclei are stained with DAPI. Images obtained at 400X magnification, scale bars, 20 μm.

Supplementary Figure 9

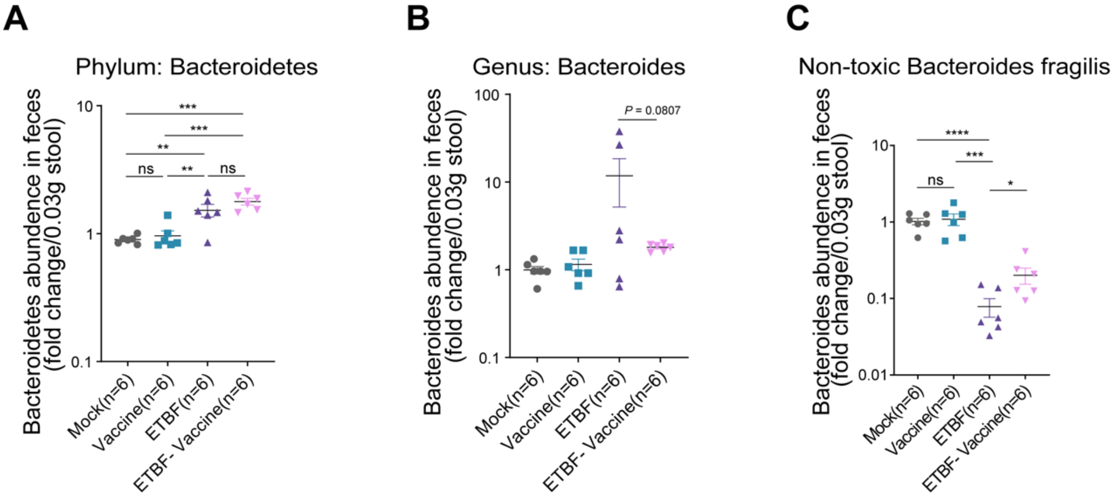

Supplementary Figure 9: Effect of vaccine and ETBF infection on the abundance of Bacteroidetes and Bacteroides and NTBF abundance in fecal DNA after boost. (A-C) qPCR was performed with Bacteroidetes, Bacteroides, and NTBF-specific primers on the stool DNA after boost 5 days (day 40). The abundance of Bacteroidetes, Bacteroides, and NTBF in bacterial DNA extracted from feces was normalized with pan-bacterial primers of the 16S rRNA gene (UNI 16S). *P* value was determined by two-tailed unpaired t test [(A) and (C)] and one-tailed unpaired t test (B). Data are mean ± SEM. **P* < 0.05, ***P* < 0.01, ****P* < 0.001, *****P* < 0.0001, ns, no significance. Data are mean ± SEM.

Supplementary Figure 10:

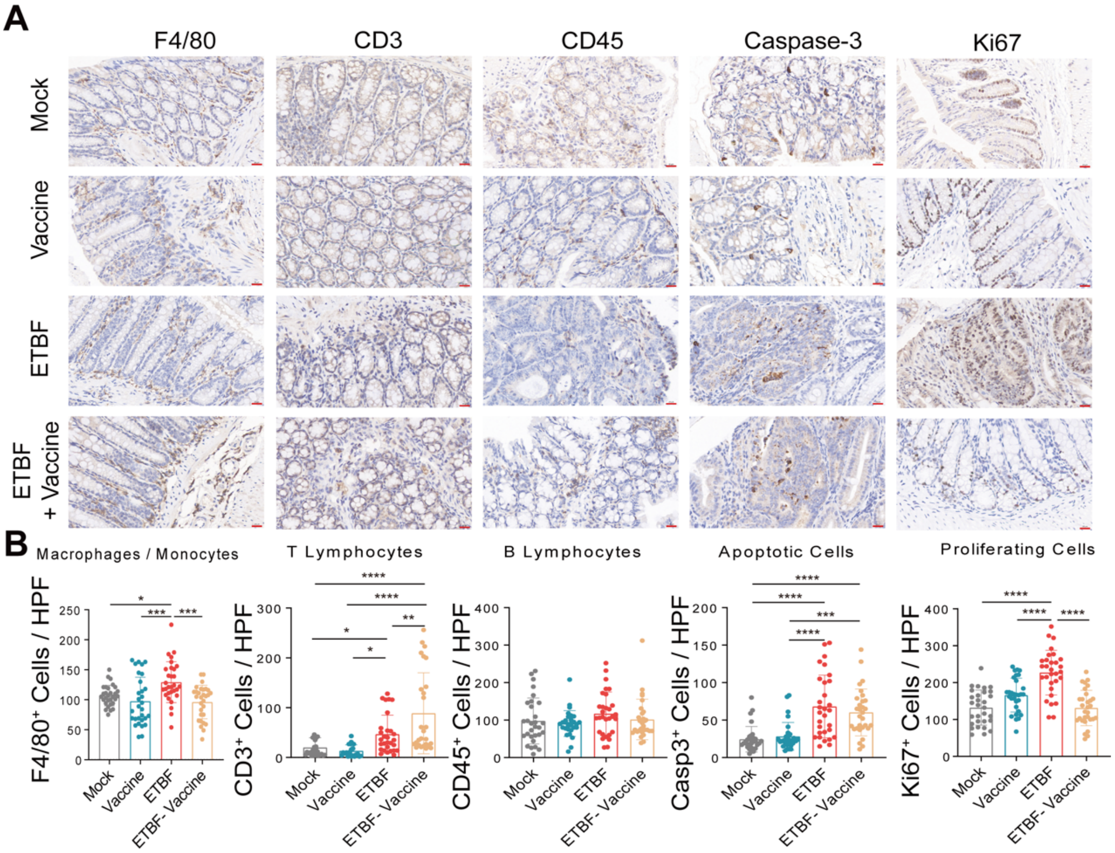

Supplementary Figure 10: The effect of the ETBF vaccine and ETBF infection on lymphocyte and cell apoptosis and proliferation in the therapeutic model. (A) Representative IHC images of T lymphocytes (CD3+), B lymphocytes (CD45+), colonic epithelial apoptotic cells (positive for caspase 3, Casp3), macrophages, monocytes (F4/80+) and proliferating/regenerating cells (positive for Ki67) staining in mice. Scale bar, 20μm. (B) Percentage of T lymphocytes (CD3+), B lymphocytes (CD45+), colonic epithelial apoptotic cells (positive for caspase 3, Casp3), macrophages and monocytes (F4/80+) and proliferating/regenerating cells (positive for Ki67) from mock, vaccine, ETBF and ETBF-Vaccine mice; n = 3 mice/group. Dot blots show corresponding quantitative analysis of CD3, CD45, Caspase-3, F4/80, and Ki67 staining. Each dot represents an HPF. Data from two independent experiments are represented as the mean ± SEM Specific n numbers are indicated in the figure. *P* values were calculated by one-way ANOVA with Holm–Sidak for multiple comparisons, **P* < 0.05, ***P* < 0.01, ****P* < 0.001, *****P* < 0.0001, ns, no significance. Error Bars represent mean ± SEM.

Supplementary Figure 11

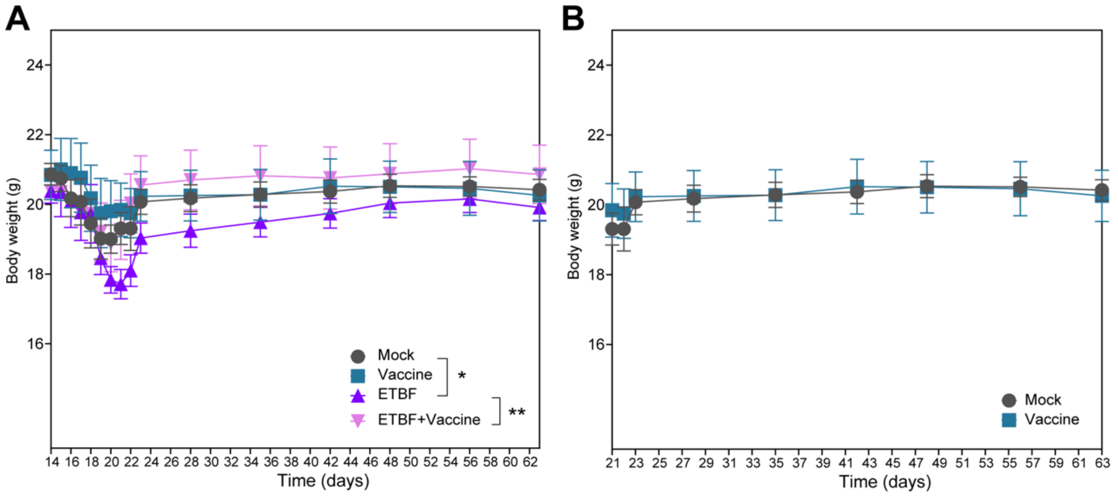

Supplementary Figure 11: The ETBF vaccine does not affect the body weight of mice. (A) Body weights of mice from day 14 to day 63 during the experiments. (B) Body weights of mice in the mock and vaccine groups from day 21 to day 63. Data are representative of two independent experiments. From day 14 to day 19, all mice were given DSS water treatment. On day 21 and day 35, the mice in the vaccine group and the ETBF+Vaccine group were administered intraperitoneal injections of the vaccine.

Supplementary Figure 12

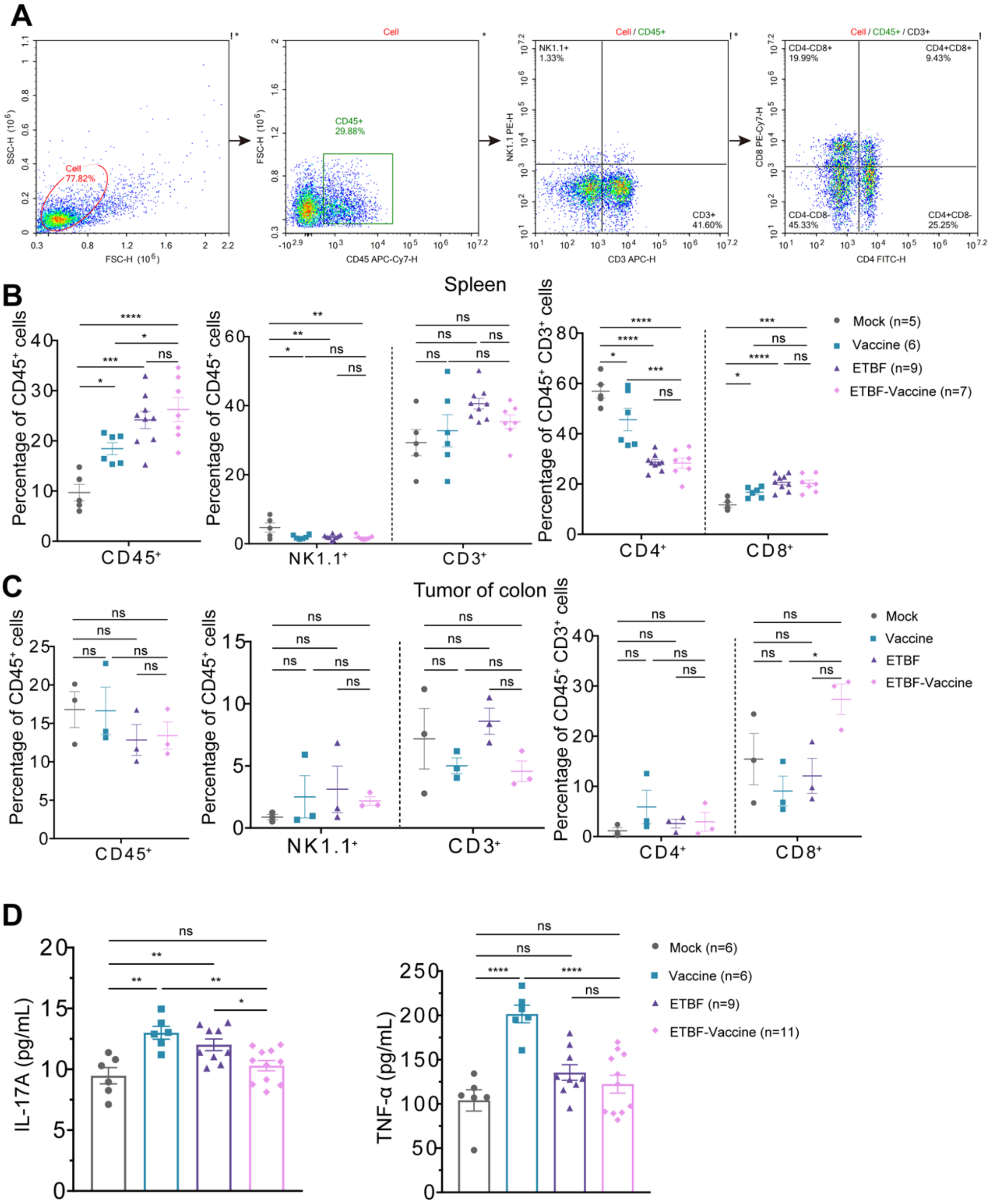

Supplementary Figure 12: Effects of ETBF inactivated vaccine on spleen and tumor lymphocyte, and pro-cancer cytokines in mice. (A) Gating strategy for FACS analysis of spleen and tumor tissue samples of mice. (B-D) Flow analysis of lymphocyte subsets in spleen samples of mice. NK and CD3⁺ cells were gated on live CD45⁺ cells. CD4⁺ CD8⁻ and CD4⁻ CD8⁺ cells were gated on live CD45⁺ CD3⁺ cells. (E-G) Flow analysis of lymphocyte subsets in colorectal tumor tissue samples of mice. (H-I) ELISA analysis was performed to detect the serum level of IL17A (left) and TNF-α (right) in mice. Data from two

independent experiments are represented as means ± SEM. *P* values were calculated by one-way ANOVA [(B), (C) and (D)] with Holm–Sidak for multiple comparisons, $*P < 0.05$, $**P < 0.01$, $***P < 0.001$, $****P < 0.0001$, ns, no significance.

Supplementary Figure 13

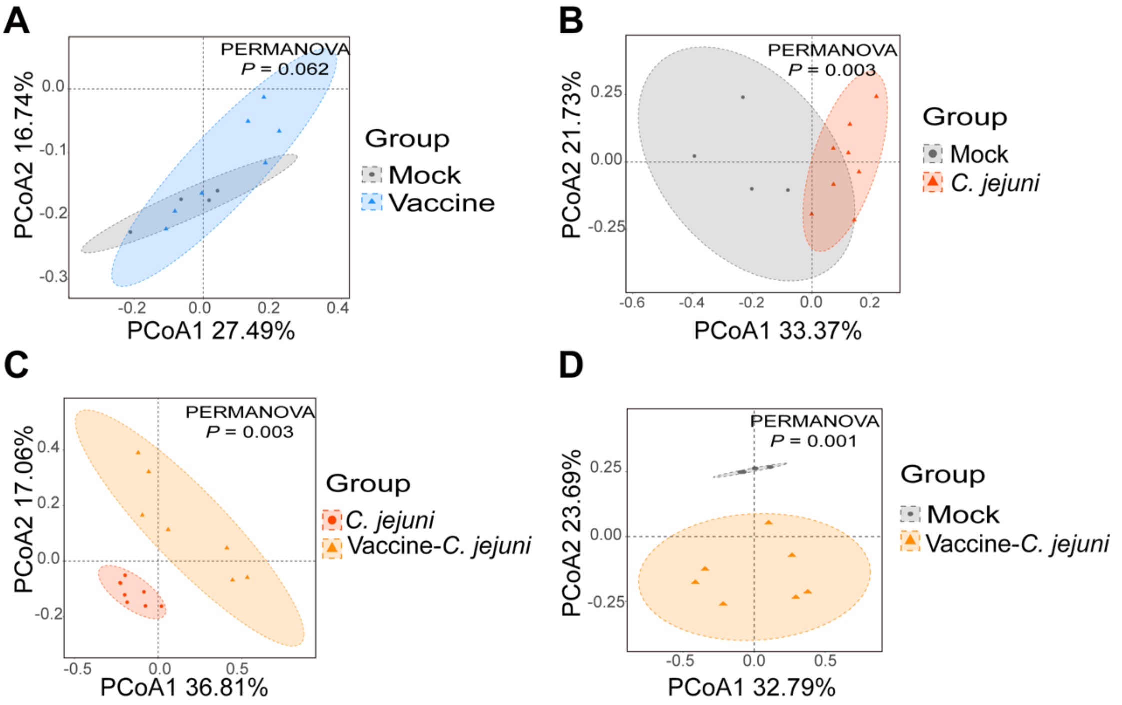

Supplementary Figure 13: Effects of inactivated *C. jejuni* vaccine on microbial composition in *Apc*$^{min/+}$DSS CRC mice by PCOA analysis. (A) PCoA analysis of the microbiota composition between the mock and the vaccine group. (B) PCoA analysis of the microbiota composition between the mock and the *C. jejuni* group. (C) PCoA analysis of the microbiota composition between the *C. jejuni* and the vaccine-*C. jejuni* group. (D) PCoA analysis of the microbiota composition between the mock and the vaccine-*C. jejuni* group.

Supplementary Figure 14

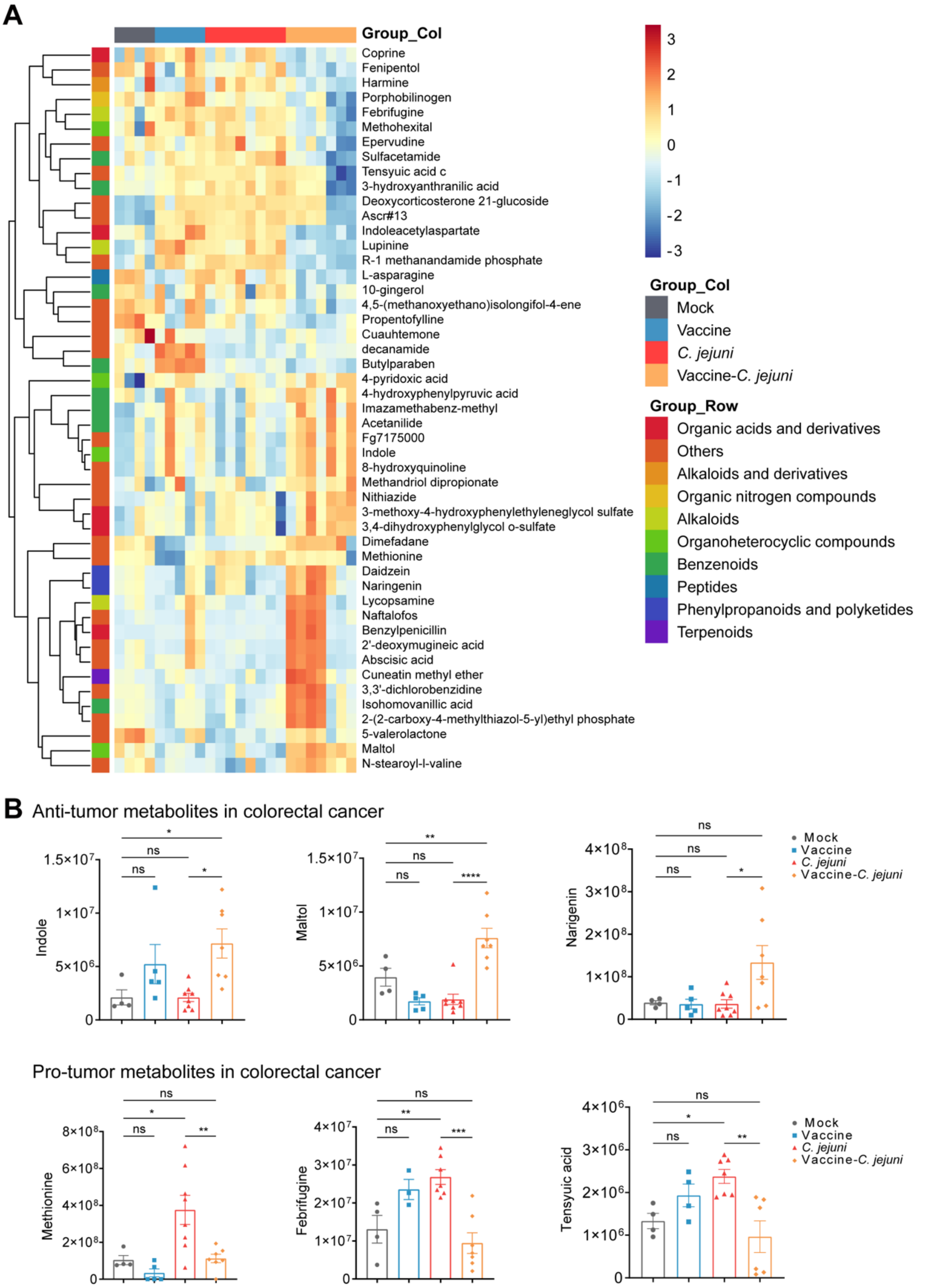

Supplementary Figure 14: Effect of inactivated vaccine on fecal metabolites in *Apc*$^{min/+}$DSS CRC mice. (A) Heatmap of the fecal metabolites from mock, vaccine, *C. jejuni*, and vaccine-*C. jejuni* group. (B) Pro-tumor and Anti-tumor metabolites in the mock, vaccine, *C. jejuni*, and vaccine-*C. jejuni* group. *P* values were calculated by one-way

ANOVA with Holm–Sidak for multiple comparisons, *$P < 0.05$, **$P < 0.01$, ***$P < 0.001$, ****$P < 0.0001$, ns, no significance. Data are mean ± SEM.

Supplementary Figure 15

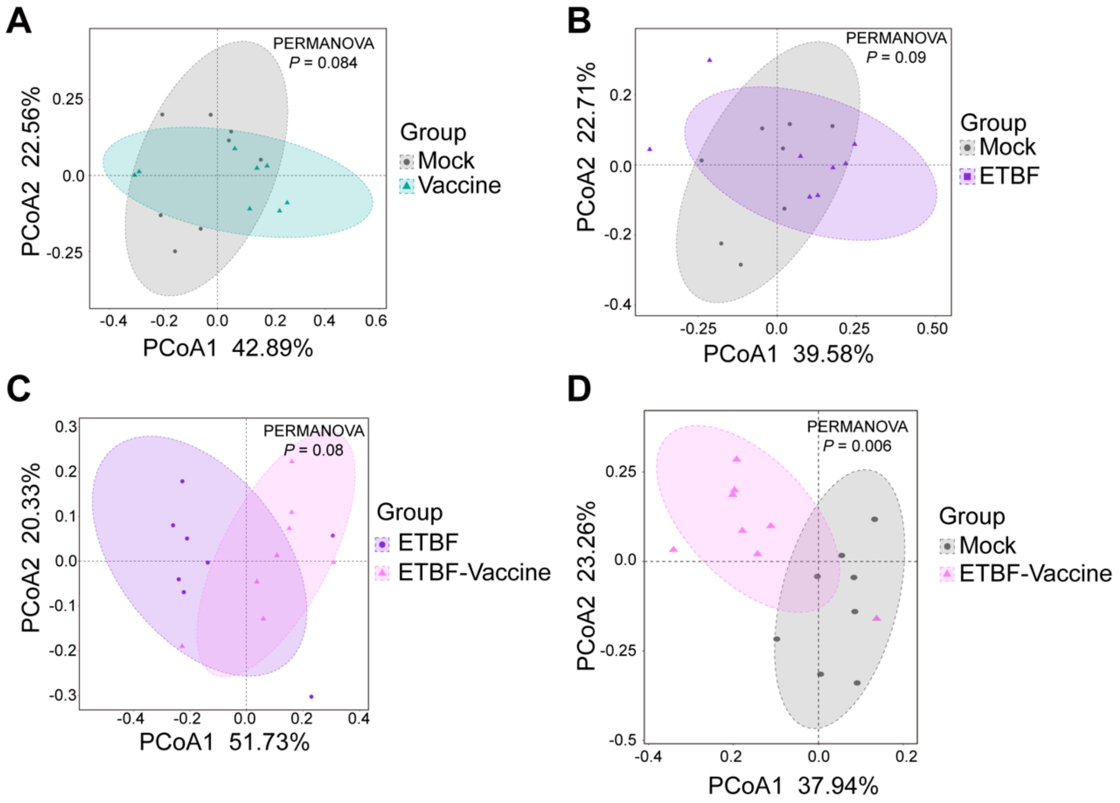

Supplementary Figure 15: Effects of inactivated ETBF vaccine on microbial composition in *Apc*$^{min/+}$DSS CRC mice by PCOA analysis. (A-D) PCoA analysis of the microbiota composition. (A) mock and vaccine group. (B) mock and ETBF group. (C) ETBF and ETBF-Vaccine group. (D) mock and ETBF-Vaccine group.

Supplementary Figure 16

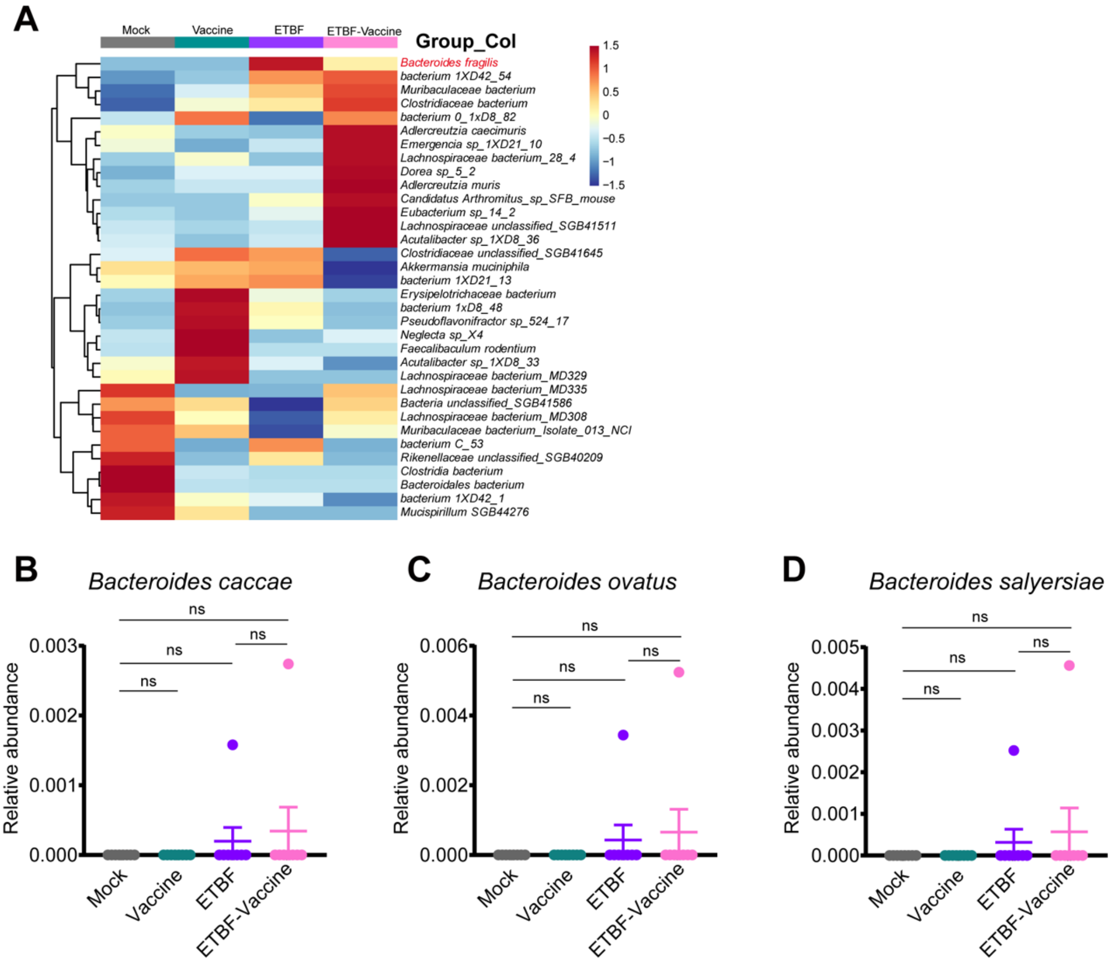

Supplementary Figure 16: Effects of inactivated ETBF vaccine on the abundance of *Bacteroides* by metagenomic sequencing analysis in *Apc*$^{min/+}$DSS CRC mice. (A) Heat map of differentially enriched bacteria at the species level in gut microbes of mice. (B) Comparison of the abundance of *Bacteroides caccae*. (C) Comparison of the abundance of *Bacteroides ovatus*. (D) Comparison of the abundance of *Bacteroides salyersiae*. *P* values were calculated by one-way ANOVA [(B), (C) and (D)] with Holm–Sidak for multiple comparisons, **P* < 0.05, ***P* < 0.01, ****P* < 0.001, *****P* < 0.0001, ns, no significance.

Supplementary Figure 17

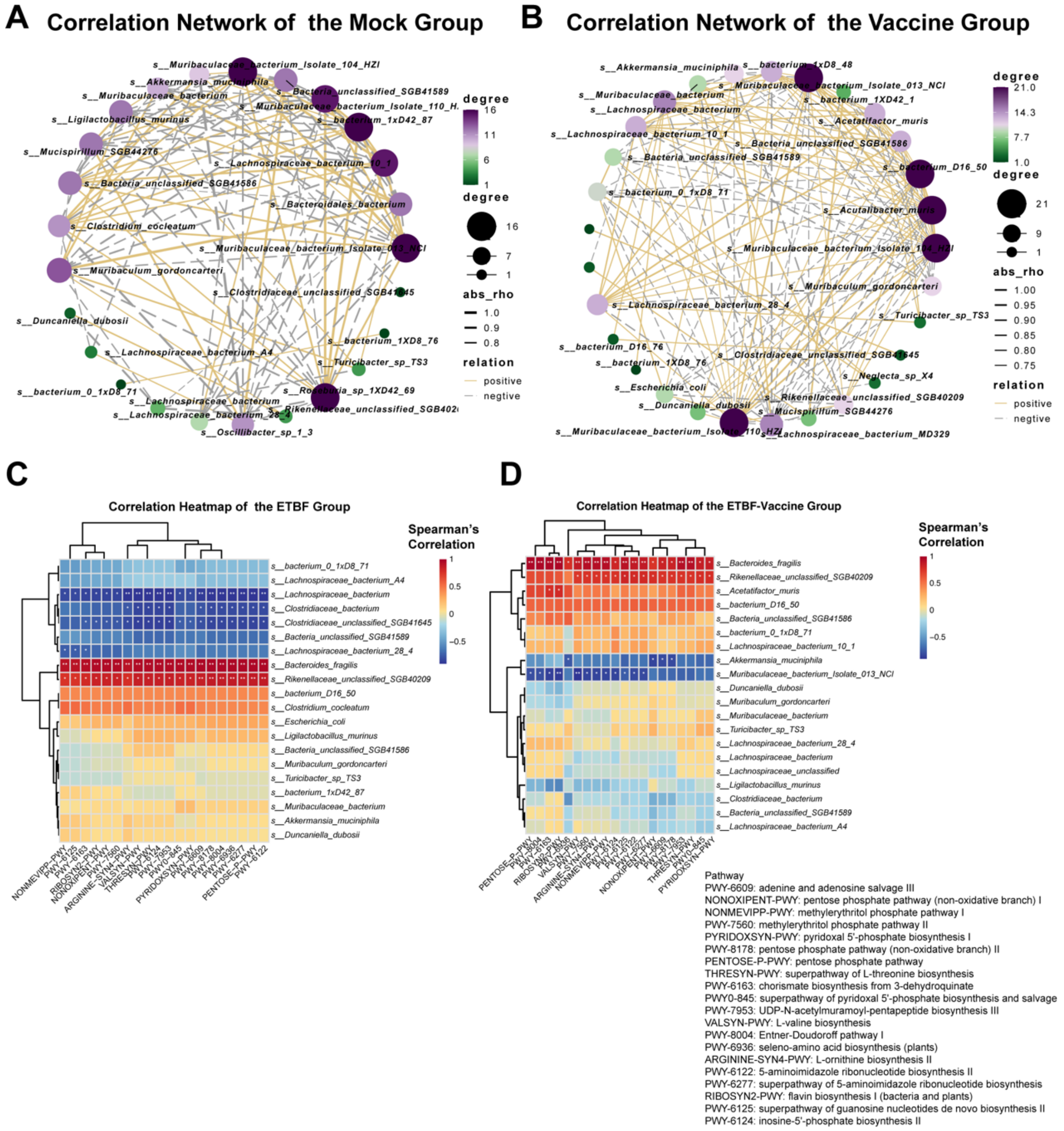

Supplementary Figure 17: Effects of inactivated ETBF vaccine on the coexistence analysis of microflora and the correlation analysis of *B. fragilis*-related functional pathways and differential species. (A-B) Correlation Network of intestinal microflora from mock and vaccine mice. (C-D) Effect of inactivated ETBF vaccine on the functional pathway of *B. fragilis* in mice. (C) Spearman correlation heatmap of the functional pathway of *B. fragilis* and differential species from the ETBF group. (D) Spearman correlation heatmap of the functional pathway of *B. fragilis* and differential species from the ETBF-Vaccine group.
